# Synthesis of a Hydroxy-15-Azasterol

**DOI:** 10.1021/acsomega.4c09907

**Published:** 2025-01-31

**Authors:** Caleb A. H. Jones, Bruce J. Melancon, Craig W. Lindsley

**Affiliations:** † Warren Center for Neuroscience Drug Discovery, Vanderbilt University, Nashville, Tennessee 37232, United States; ‡ Department of Pharmacology, Vanderbilt University School of Medicine, Nashville, Tennessee 37232, United States; § Department of Chemistry, Vanderbilt University, Nashville Tennessee 37232, United States

## Abstract

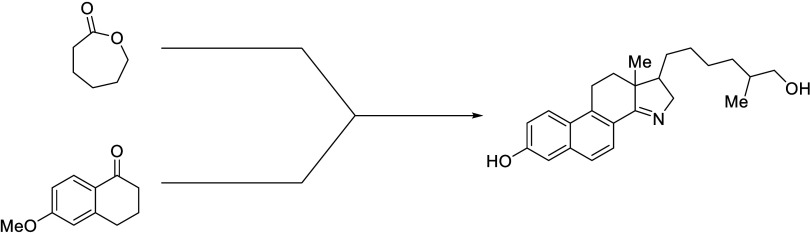

Niemann-Pick type C (NPC) is a lysosomal storage disorder that will cause eventual brain damage with limited treatment options available. Though clinical trials are undergoing with repurposed pharmaceuticals, no novel chemotype exists purely for the treatment of NPC. In 2021, an azasterol was found to bind to both NPC1 and NPC2 proteins and is considered as a cholesterol mimic. A convergent synthesis to obtain a hydroxy-15-azasterol was completed in 12 steps, from 2-oxepanone.

## Introduction

**Figure 1:**
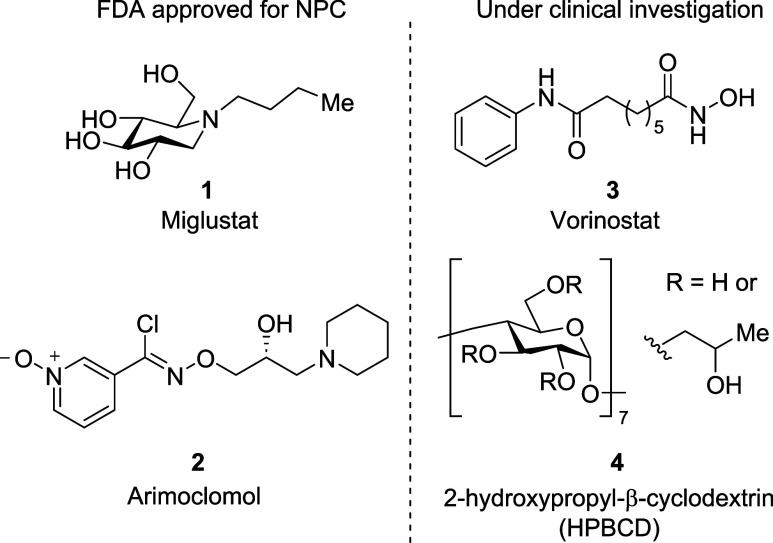
Repurposed medications for the treatment of NPC: Miglustat, Arimoclomol, Vorinostat, and HPBCD.

Niemann-Pick type C (NPC) is a lysosomal storage disorder that affects the body’s ability to transport and manage the cholesterol and lipids within the cells due to mutations in NPC1 or NPC2 genes.^1,2^ This buildup of lipids can cause extensive brain damage that complicates head movement, walking, swallowing, an eventual loss of vision and hearing, as well causing cognitive and psychological complications.^3−5^ No cure is available for NPC with the only FDA approved treatment being a combination of miglustat and arimoclomol (**1** and **2**, Figure 1).^6−9^

Additional compounds that are currently undergoing clinical trials are vorinostat and 2-hydroxypropyl-β-cyclodextrin (HPBCD) (**3** and **4**, Figure 1).^8,10,11^ Of these compounds, HPBCD is the most studied and has the most promising prognosis. However, HPBCD is not brain penetrant and requires intrathecal injection. Additionally, outcomes are dependent on the disease state upon initial treatment, and hearing loss has been observed in animal studies.^12−15^ It is also worth noting that these current standards of care are repurposed molecules from other primary indications, and a need exists for the development of novel scaffolds for NPC1 and NPC2 protein binding.

**Figure 2:**
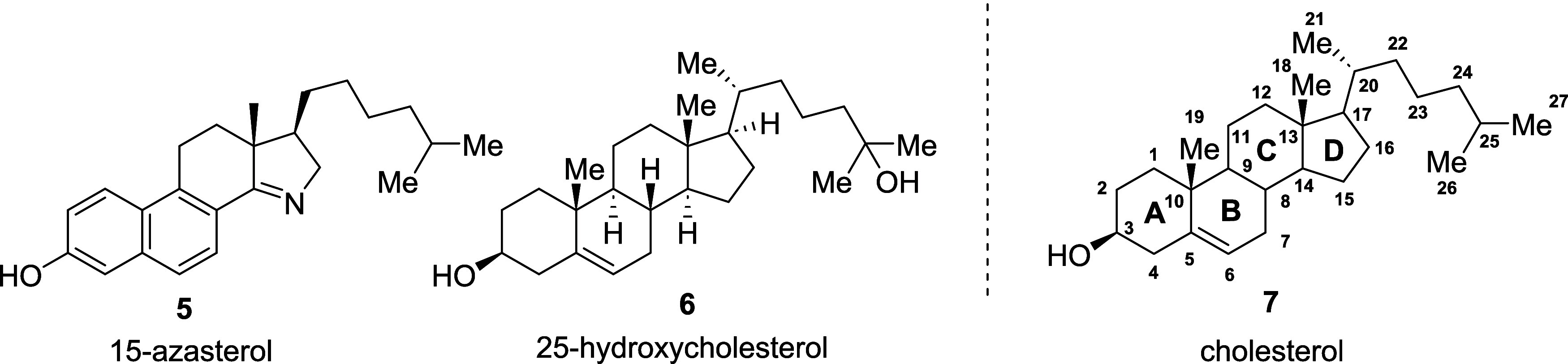
15-azasterol cholesterol mimic, 25-hydroxycholestrol, and annotated cholesterol.

Since 2021, 15-azasterol (**5**) (Figure 2) has been studied as a biochemical tool for understanding cholesterol trafficking. Within this study the authors also used molecular docking of **5** in NPC1 and NPC2 proteins and found there was a significant overlay between cholesterol **7** and **5**.^16^ This suggests that NPC1 and NPC2 could be targeted using 15-azasterols as long as they mimic a cholesterol binding motif.

However, oxysterols have been found to bind tighter and re-enforce the stability of NPC, suggesting **5** could potentially be improved with a hydroxyl group on the lipophilic tail.^17,18^ We therefore were interested in developing a synthesis to install a hydroxy group on **5** to mimic 25-hydroxycholesterol (**6**, Figure 2). Herein we describe the synthesis of a hydroxy-15-azasterol (**8**)

## Results and Discussion

**Figure 3:**
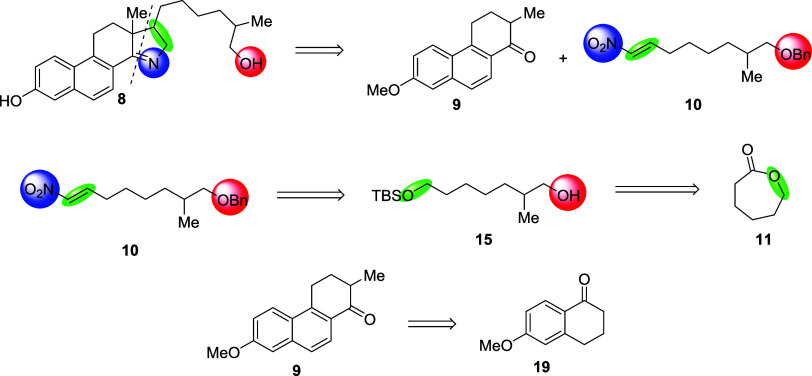
Retrosynthetic analysis of Hydroxy-15-azasterol (**8**).

Our retrosynthetic strategy is shown in Figure 3, and was inspired by the work of Weist and co-workers.^16^ The synthesis of the hydroxy-15-azasterol (**8**) was divided into two segments: a precursor of the hydroxylated hydrophobic tail (**10**) and the central tetrahydrophenanthrenone core (**9**). The two segments are designed to react through a Michael addition of a nitroalkene such that the D ring of the azasterol could form via a reductive condensation. Formation of **9** has been previously reported,^19^ however we modified the synthesis such that most steps can be performed in an open atmosphere. The central core (**9**) was derived from the tetralone (**19**) where the remainer of the skeleton could be synthesized through homologation, and condensation reactions. The nitroalkene (**10**) had to be synthesized while avoiding cross-reactivity of other functionalities present in the molecule. This led us to the monoprotected diol (**15**) which allowed for selective manipulation of the 7-heptanol while maintaining the hydroxy at the 1 position. To obtain the initial heptanol we used 2-oxepanone (**11**) which upon ring opening via a Grignard reaction would give the correct carbon skeleton and a ketone as a functional handle.

**Scheme 1:**
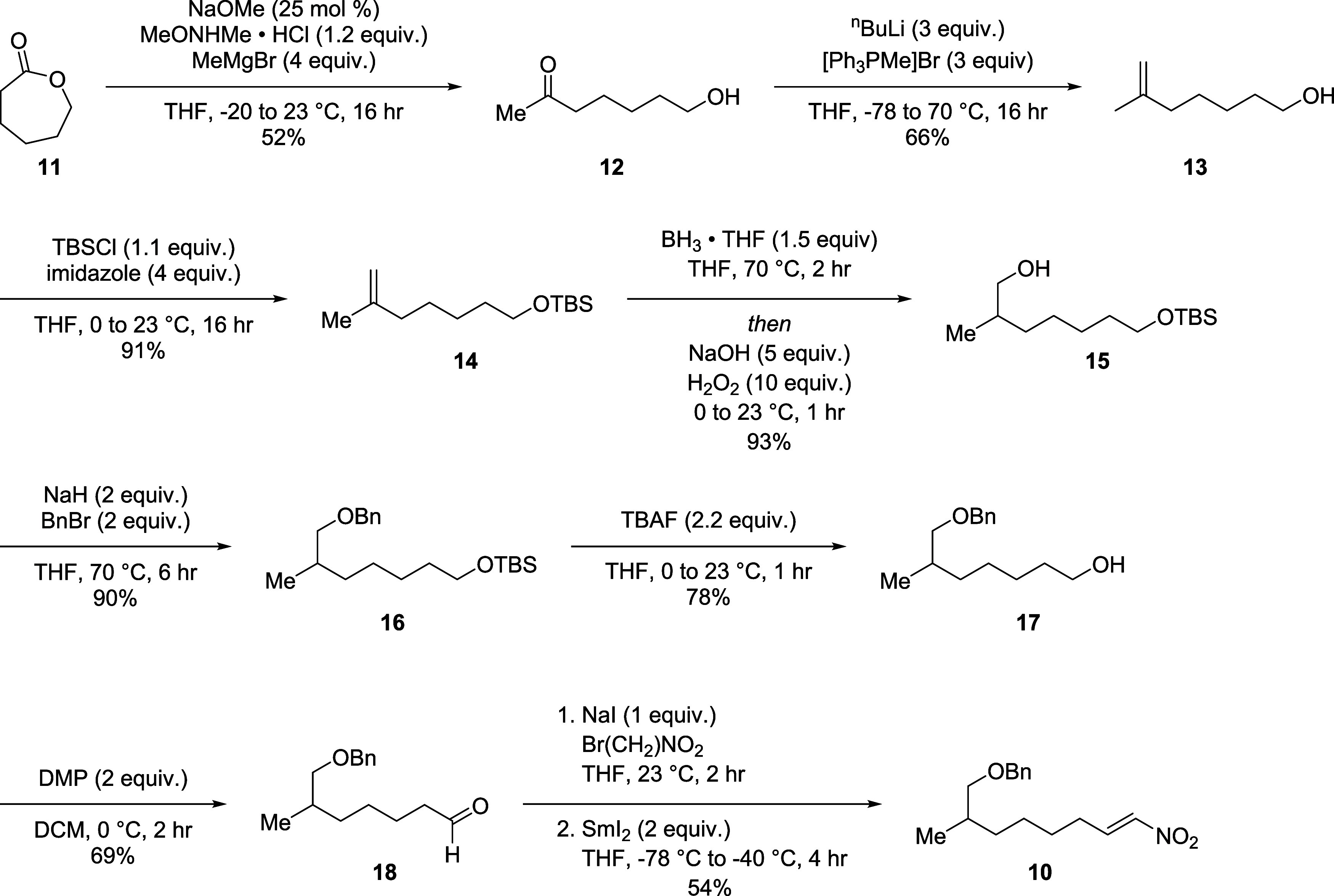
Synthesis of Nitroalkene 10

The synthesis of the hydroxylated hydrophobic tail precursor is outlined in Scheme 1. A one-pot Weinreb amidation-Grignard reaction of 2-oxepanone **11** was performed to obtain the hydroxy ketone **12** in 52% yield with the major side product being over alkylation to the tertiary alcohol. From the ketone, terminal alkene **13** was synthesized via a Wittig reaction with [MePPh_3_]Br followed by TBS protection of the alcohol to give **14**. We then oxidized the olefin via hydroboration-oxidation to obtain the mono-TBS protected diol **15** in 93% yield. Protection of **15** with benzyl bromide followed by the removal of the TBS group with TBAF yielded **17**, which was then oxidized with Dess–Martin periodinane to give the aliphatic aldehyde **18**. Upon isolation, the aldehyde was directly subjected to the nitroalkene formation the same day to prevent auto-oxidation to the carboxylic acid. No desired product was observed after subjecting **18** under classical Henry reaction conditions and dehydration^20^ nor when catalyzed by piperidine.^21^ However, adapting a synthesis described by Concellon et al.^22^ we found a Henry reaction with sodium iodide and bromo-nitromethane followed by an elimination with fresh SmI_2_ gave the desired nitroalkene **10**.

**Scheme 2:**
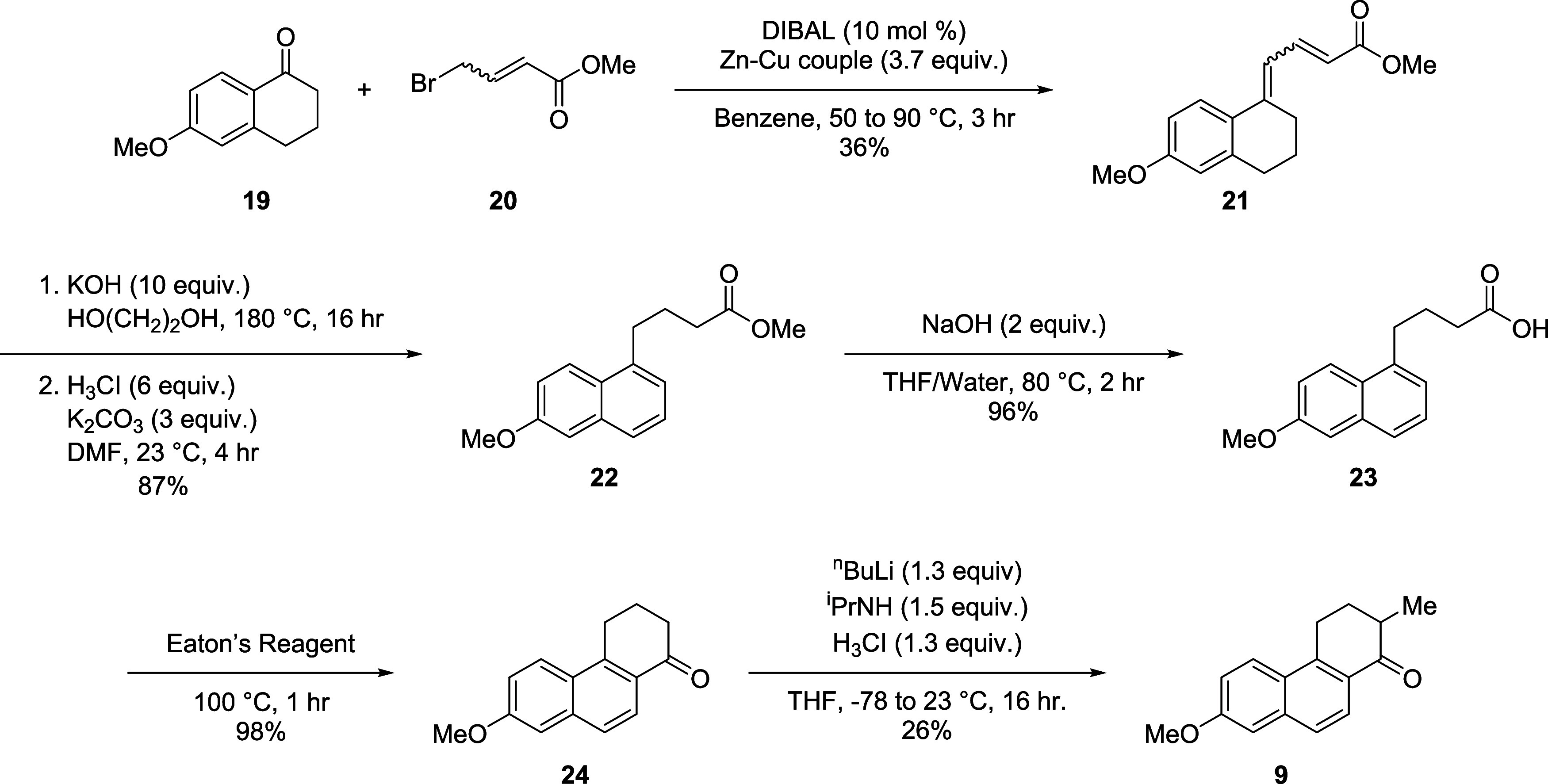
Synthesis of Phenanthrenone 9

The tetrahydrophenanthrenone core (**9**, Scheme 2) was synthesized starting from a Barbier condensation between 4-bromocrotonate **20** and tetralone 1**9**. Rigorous reaction conditions were necessary for the reaction to proceed; DIBAL was necessary to activate the zinc–copper coupling at 50 °C, followed by the slow addition of **19** and **20** in benzene at 80 °C. Higher yields can also be obtained through the addition of excess **20** portion wise at 90 °C over the course of 1 h. Olefin isomerization at high temperatures under strongly basic conditions were found to be the best and most consistent route to obtain the naphthalene core. This method also hydrolyzed the ester which was suitable for the cyclization step to the phenanthrenone core **24**. However, partial demethylation of the phenol was also observed giving a mixture of both the 6-methoxy and 6-hydroxy products. Methylation with iodomethane was necessary prior to condensation with Eaton’s reagent as the 6-hydroxy forms unfavorable side products and greatly complicates purification giving **22**. Ester hydrolysis (of **22**) to the carboxylic acid was carried out with sodium hydroxide in tetrahydrofuran (THF) before cyclizing into the naphthalene ring using Eaton’s reagent. After cyclization, α-methylation of ketone **24** was accomplished using freshly generated LDA and iodomethane. Although the reaction proceeds in low yield (26%), **24** can be recovered up to 20%.

**Scheme 3:**
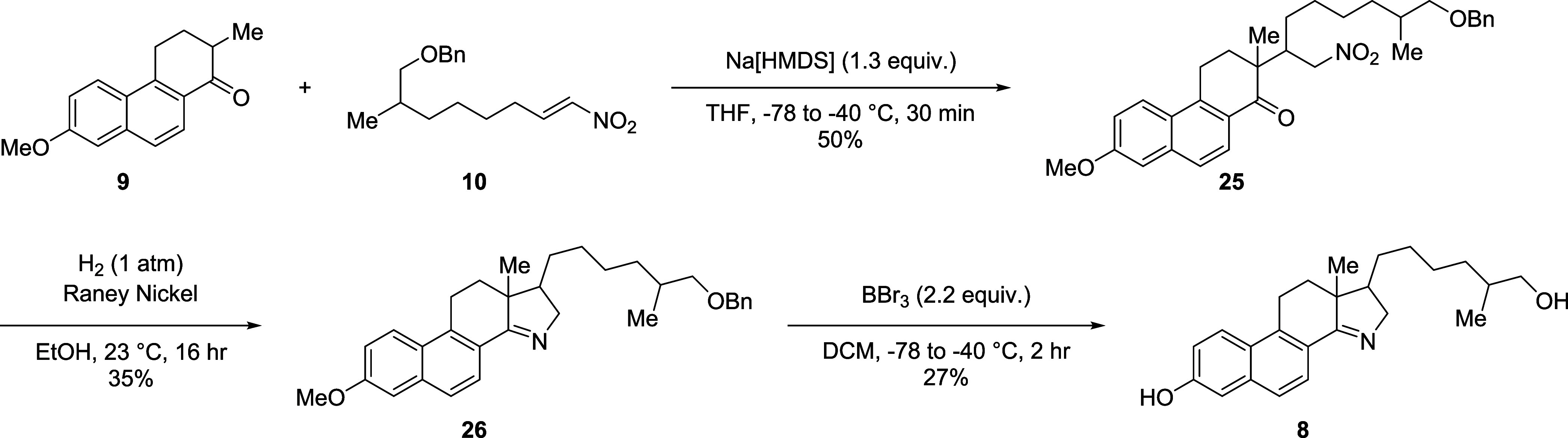
Synthesis of Hydroxy-15-Azasterol (8) from 9 and 10

Once the phenanthrenone core **9** and the nitroalkene **10** were prepared, a Michael addition was performed to give **25** as a complex mixture of diastereomers which were not separable by chromatography (Scheme 3). The diastereomeric mixture of **25** was then subjected to a one-pot reductive condensation under an atmosphere of hydrogen with catalytic Raney nickel to give the protected 15-azasterol **26**. It was at this step that a major set of diastereomers were isolated as a mixture. Interestingly, the benzyl group on the primary alcohol was not removed during the hydrogenation, though it was easily removed by BBr_3_ in the following step. The benzyl protecting group is rapidly removed at −78 °C, however demethylation of the phenol requires warming to −40 °C. Unfortunately, **26** also undergoes an Appel-type side reaction at this temperature, displacing the primary alcohol to give an alkyl bromide as the major side product. A benzylated variant of **22** was synthesized to avoid this side reactivity, however upon cyclization with Eaton’s reagent, the 6-benzyloxy is debenzylated and mesylated complicating purification, drastically reducing yields.

Overall, our synthesis of **8** employed a convergent route wherein key nitroalkene **10** was synthesized in 8% yield over 9 steps from 2-oxepanone (**11**) and core phenanthrenone (**9**) was synthesized in 8% over 6 steps from 6-methoxytetralone **10** before converging for the synthesis of **8**, which was obtained as a set of diastereomers in 5% yield over 3 steps from **9** and **10**. Additionally it is worth noting that when analyzing all 8 stereoisomers of **8** via chiral SFC only two peaks where observed as two groups of diastereomers with a ratio of 55:45 (see Supporting Information for chromatogram).

## Conclusions

In this report we describe the synthesis of a hydroxy-15-azasterol (**8**) starting from a commercial feedstock with the majority of the steps open to air and limited precautions to avoid moisture. Sterols themselves are complex, diverse, biologically relevant molecules and challenging to synthesize de novo. Furthermore, incorporation of a nitrogen atom adds a layer of complexity to this class of molecules by just synthetic standards. Future endeavors should focus on improving the diastereoselectivity at key steps (synthesis of **15** and **25**). If stereoselectivity cannot be obtained during the synthesis, then it could be beneficial to use chiral chromatography for **16** and **26** specifically as chiral separation for **8** is ineffective.

## Experimental Section

### General Considerations

Syntheses and manipulations were conducted in air unless otherwise specified. Reaction solvents were purchased from Sigma-Aldrich in anhydrous form with a sure seal. All reagents and building blocks for which procedures are not given below were procured from commercial vendors and used without further purification. ^1^H, ^13^C{^1^H}, and two-dimensional nuclear magnetic resonance (2D NMR) spectra were recorded on a 400 MHz Bruker AV-400 spectrometer at ambient temperature unless otherwise noted.^1^H and ^13^C{^1^H} chemical shifts are referenced to residual solvent signals in the following solvents: CDCl_3_ (^1^H NMR: 7.26 ppm; ^13^C{^1^H} NMR: 77.16 ppm) and DMSO-*d*_6_ (^1^H NMR: 2.50 ppm, ^13^C{^1^H} NMR: 39.52 ppm). Chemical shifts are reported in ppm and multiplicities are abbreviated as follows: br = broad, s = singlet, d = doublet, t = triplet, q = quartet, quint = quintet, o = octet, dd = doublet of doublets, dt = doublet of triplets, td = triplet of doublets, ddd = doublet of doublet of doublets, m = multiplet. Automated flash column chromatography (normal phase) was conducted using a Teledyne ISCO CombiFlash system with certified ACS grade solvents. Reverse phase HPLC was performed on a Gilson preparative reverse-phase HPLC system comprised of a 333 aqueous pump with solvent-selection valve, 334 organic pump, GX-271 or GX-281 liquid hander, two column switching valves, and a 155 ultraviolet (UV) detector. UV wavelength for fraction collection was user-defined, with absorbance at 254 nm always monitored. Column: Phenomenex Axia-packed Gemini C18, 30 × 50 mm^2^, 5 μm. Mobile phase: CH_3_CN in H_2_O (0.05% v/v NH_4_OH). Gradient conditions: 0.75 min equilibration, followed by user-defined gradient (starting organic percentage, ending organic percentage, duration), hold at 95% CH_3_CN in H_2_O (0.05% v/v NH_4_OH) for 1 min, 50 mL/min, 23 **°**C.

### Analytical Chiral SFC Separation

Chiral SFC separation was performed on a Thar (Waters) Investigator. Column: Chiral Technologies Phenomenex Lux-Cellulose 3, 4.6 × 250 mm^2^, 5 um. Gradient conditions: 15% MeOH/0.1% DEA (isocratic) in CO_2_ over 7 min, hold at 50% CO_2_ for 1 min. Flow rate: 3.5 mL/min. Column temperature: 40**°** C. System backpressure: 100 bar.

### Mass Spectrometry

High-resolution mass spectra were obtained on an Agilent 6540 UHD Q-TOF with Dual AJS source. MS parameters were as follows: fragmentor: 150; capillary voltage: 4000 V; nebulizer pressure: 60 psi; drying gas flow: 13 L/min; drying gas temperature: 275 °C.  Samples were introduced via an Agilent 1290 ultrahigh performance liquid chromatography (UHPLC) comprised of a G4220A binary pump, G4226A ALS, G1316C TCC, and G4212A DAD with ULD flow cell.  UV absorption was observed at 215 and 254 nm with a 4 nm bandwidth.  Column:  Waters Acquity BEH C18, 1.0 × 50 mm^2^, 1.7 um.  Gradient conditions: 5–95% CH_3_CN in H_2_O (0.1% Formic Acid) over 1.25 min, hold at 95% CH_3_CN for 0.25 min, 0.3 mL/min, 40 °C.

### Experimental Procedures

#### 7-Hydroxyheptan-2-one (**12**)

To an oven-dried 500 mL round-bottom flask equipped with a stir bar and an addition funnel was added 2-oxepanone (3.9 mL, 35.04 mmol, 1.0 equiv), *N*,*O*-dimethylhydroxylamine hydrochloride (4.1 g, 42.05 mmol, 1.2 equiv), and sodium methoxide (0.47 g, 8.76 mmol, 25 mol %), which were dissolved in THF (200 mL). The reaction mixture was then cooled to −20 °C with a 70% water–methanol (v/v) dry ice bath followed by the dropwise addition of methylmagnesium bromide (3.0 M in diethyl ether, 46.7 mL, 140.18 mmol). The solution was warmed slowly to room temperature (via the slurry melting) and stirred overnight. The following morning, the reaction was quenched with 4 N HCl (100 mL), and the organic and aqueous layer were separated. The aqueous portion was then extracted with ethyl acetate (×2). Combined organics were then washed with brine, dried over anhydrous Na_2_SO_4_, filtered, and made concentrated. Crude product was purified using a Teledyne ISCO Combi-Flash system (solid loading, 80G column, 0–100% EtOAc in Hexanes, 30 min run, product) to give **12** (2.39 g, 18.36 mmol, 52% yield) as a clear oil. Spectroscopic data matched previous reports.^23^

^1^H NMR (400 MHz CDCl_3_) δ 3.65 (t, *J* = 6.5 Hz, 2H), 2.43 (t, *J* = 7.3 Hz, 2H), 2.14 (s, 3H), 1.54–1.64 (m, 4H), 1.34–1.40 (m, 2H).

#### 6-Methylhept-6-en-1-ol (**13**)

In a 500 mL oven-dried round-bottom flask under a stream of nitrogen, [MePPh_3_]Br (18.43 g, 51.44 mmol, 3.0 equiv) was dissolved in THF (138 mL) and cooled to −78 °C followed by the dropwise addition of *n*-butyllithium (2.5 M in hexanes, 20.6 mL, 51.44 mmol, 3.0 equiv). Upon complete addition, the reaction mixture was warmed to room temperature briefly before cooling to 0 °C for the addition of **12** (2.23 g, 17.15 mmol, 1.0 equiv) in THF (10 mL). The mixture was stirred at this temperature for 10 min before heating to 70 °C and stirring overnight. The following morning, the reaction was cooled to 0 °C, quenched with saturated NH_4_Cl, and extracted with ethyl acetate (×2). Combined organics were then dried over anhydrous Na_2_SO_4_, filtered, and made concentrated. Crude product was purified using a Teledyne ISCO Combi-Flash system (solid loading, 120G column, 10–100% EtOAc in Hexanes, 30 min run) to give 1**3** (1.45 g, 11.30 mmol, 66% yield) as a clear oil. Spectroscopic data matched previous reports.^23^

^1^H NMR (400 MHz CDCl_3_) δ 4.68 (d, *J* = 11.6 Hz, 2H), 3.65 (t, *J* = 6.6 Hz, 2H), 2.02 (t, *J* = 7.5 Hz, 2H), 1.71 (s, 3H), 1.55–1.62 (m, 2H), 1.43–1.50 (m, 2H), 1.32–1.39 (m, 3H).

#### *tert*-Butyldimethyl((6-methylhept-6-en-1-yl)oxy)silane (**14**)

In an oven-dried flask, 1**3** (820 mg, 6.40 mmol, 1.0 equiv) was dissolved in THF (14 mL), followed by the addition of imidazole (1.74 g, 25.58 mmol, 4.0 equiv). Once the solution was homogeneous, *tert*-butyldimethylchlorosilane (1.06 g, 7.04 mmol, 1.1 equiv) in THF (14 mL) was added, and the reaction was stirred overnight at room temperature. Upon completion, the reaction was quenched with 1 N HCl (30 mL) and extracted with ethyl acetate (3 × 25 mL). Combined organics were then washed with brine, dried over anhydrous Na_2_SO_4_, filtered, and made concentrated. **14** (1.41 g, 5.81 mmol, 91% yield) was obtained as a clear oil and taken forward without further purification.

^1^H NMR (400 MHz CDCl_3_) δ 4.67 (d, *J* = 9.8 Hz, 2H), 3.60 (t, *J* = 6.2 Hz, 2H), 2.01 (t, *J* = 7.5 Hz, 2H), 1.71 (s, 3H), 1.40–1.56 (m, 4H), 1.29–1.35 (m, 2H), 0.89 (s, 9H), 0.05 (s, 6H); ^13^C{^1^H} NMR (101 MHz CDCl_3_) δ 146.3,105.8,63.4,38.0,32.9,27.6,26.1,25.7,22.5,18.5,–5.1; Note: compound does not ionize in either negative or positive modes in the HRMS.

#### 7-((*tert*-Butyldimethylsilyl)oxy)-2-methylheptan-1-ol (**15**)

In an oven-dried round-bottom flask, **14** (1.41 g, 5.81 mmol, 1.0 equiv) was dissolved in THF (11.5 mL) and cooled to 0 °C followed by the addition of borane tetrahydrofuran complex (1 M in THF, 8.7 mL, 8.71 mmol, 1.5 equiv). The mixture was heated to 70 °C and stirred for 2 h. At this point NMR indicated full conversion and the reaction was cooled to 0 °C before the slow addition of 2 M sodium hydroxide (14.5 mL, 29.03 mmol, 5.0 equiv) and hydrogen peroxide (30% aqueous solution, 5.9 mL, 58.06 mmol, 10.0 equiv) and stirred for an additional hour. The mixture was then diluted in ethyl acetate, washed with water and brine, dried over anhydrous Na_2_SO_4_, filtered and made concentrated. **15** (1.41 g, 5.40 mmol, 93% yield) was obtained as a clear oil and used without further purification.

^1^H NMR (400 MHz CDCl_3_) δ 3.59 (t, *J* = 6.5 Hz, 2H), 3.38–3.51 (m, 2H), 1.56–1.62 (m, 4H), 1.24–1.48 (m, 5H), 1.04–1.14 (m, 1H), 0.90 (d, *J* = 6.7 Hz, 3H), 0.88 (s, 9H), 0.04 (s, 6H); ^13^C{^1^H} NMR (101 MHz CDCl_3_) δ 68.5,63.4,35.9,33.3,32.3,26.9,26.3,26.1,18.5,16.7,–5.1; HRMS (ESI) calculated for C_14_H_32_O_2_Si: 261.2244 ([M + H]^+^), found 261.2245.

#### ((7-(Benzyloxy)-6-methylheptyl)oxy)(*tert*-Butyl)dimethylsilane (**16**)

In an oven-dried round-bottom flask, 1**5** (1.41 g, 5.40 mmol, 1.0 equiv) was dissolved in THF (54 mL) and treated with sodium hydride (432 mg, 10.80 mmol, 2.0 equiv). The suspension was then stirred for 15 min before the addition of benzyl bromide (1.3 mL, 10.80 mmol, 2.0 equiv). The reaction was heated to 70 °C and stirred for 6 h, at which point TLC indicated completion. The mixture was then cooled to 0 °C, quenched with water, and extracted into ethyl acetate (×3). Organics were then washed with brine, dried over anhydrous Na_2_SO_4_, filtered and made concentrated. Crude product was purified using Teledyne ISCO Combi-Flash system (liquid loading, 80G column, 0% EtOAc, for 10 min then 0–100% EtOAc in Hexanes for 20 min, collect all fractions [low UV activity], product elution ∼20%). **16** (1.71 g, 4.88 mmol, 90% yield) was obtained as a yellow oil.

^1^H NMR (400 MHz CDCl_3_) δ 7.18–7.30 (m, 5H), 4.43 (d, *J* = 1.2 Hz, 2H), 3.52 (t, *J* = 3.5 Hz, 2H), 3.14–3.27 (m, 2H), 1.68 (o, *J* = 6.5 Hz, 1H), 1.15–1.48 (m, 7H), 1.00–1.08 (m, 1H), 0.85 (d, *J* = 6.7 Hz, 3H), 0.82 (s, 9H), −0.02 (s, 6H); ^13^C{^1^H} NMR (101 MHz CDCl_3_) δ139.0, 128.4 127.7, 127.5, 76.2, 73.1, 63.4, 33.8, 33.6, 33.0, 26.9, 26.3, 26.1, 18.5, 17.3, −5.1; HRMS (ESI) calculated for C_21_H_38_O_2_Si 351.2714 ([M + H]^+^), found 351.2716.

#### 7-(Benzyloxy)-6-methylheptan-1-ol (**17**)

A round-bottom flask containing 16 (3.22 g, 9.18 mmol, 1.0 equiv) was cooled to 0 °C, and tetrabutylammonium fluoride (23 mL, 22.96 mmol, 2.5 equiv) (1 M in THF) was added. The mixture was then warmed up to 23 °C and stirred for 1 h. Upon completion, the reaction was quenched with water and extracted with ethyl acetate (×3). Combined organics were washed with brine, dried over anhydrous Na_2_SO_4_, filtered, and made concentrated. Crude product was purified using a Teledyne ISCO Combi-Flash system (liquid loading, 80G column, 0–50% EtOAc in Hexanes, 30 min run) to give **17** (1.71 g, 7.24 mmol, 78% yield) as a clear oil.

^1^H NMR (400 MHz CDCl_3_) δ 7.18–7.28 (m, 5H), 4.42 (s, 2H), 3.55 (t, *J* = 6.6 Hz, 2H), 3.21 (m, 2H), 1.69 (o, *J* = 5.4 Hz, 1H), 1.45–1.56 (m, 3H), 1.17–1.41 (m, 5H), 1.01–1.09 (m, 1H), 0.85 (d, *J* = 6.7 Hz, 3H); ^13^C{^1^H} NMR (101 MHz CDCl_3_) 138.9, 128.4, 127.7, 127.5, 76.1, 73.1, 63.1, 33.7, 33.5, 32.8, 26.8, 26.1, 17.3; HRMS (ESI) calculated for C_15_H_24_O_2_ 237.1849 ([M + H]^+^), found 237.1845.

#### 7-(Benzyloxy)-6-methylheptanal (**18**)

A solution of 1**7** (250 mg, 1.06 mmol, 1.0 equiv) in dichloromethane (5.3 mL) was cooled to 0 °C followed by the addition of Dess–Martin periodinane (897 mg, 2.12 mmol, 2.0 equiv). The reaction was then stirred at this temperature for 2 h before being diluted with ethyl ether, filtered through a polytetrafluoroethylene (PTFE) filter, and concentrated. Crude product was purified using a Teledyne ISCO Combi-Flash system (liquid loading, 24G column, 0–30% EtOAc in Hexanes, 12 min run) to give **18** (172 mg, 0.73 mmol, 69% yield) as a clear oil and used immediately in the next step (oxidizes readily to the carboxylic acid).

^1^H NMR (400 MHz CDCl_3_) δ 9.68 (t, *J* = 1.8 Hz, 1H), 7.19–7.29 (m, 5H), 4.42 (s, 2H), 3.16–3.25 (m, 2H), 2.33 (td, *J* = 11.0, 1.8 Hz, 2H), 1.65–1.73 (m, 1H), 1.51–1.59 (m, 2H), 1.17–1.44 (m, 3H), 1.03–1.11 (m, 1H), 0.85 (d, *J* = 6.7 Hz, 3H); ^13^C{^1^H} NMR (101 MHz CDCl_3_) δ203.0, 138.8, 128.4, 127.6, 127.2, 75.9, 73.1, 44.0, 33.5, 33.4, 26.6, 22.4, 17.1; HRMS (ESI) calculated for C_15_H_22_O_3_ 249.1496 ([M-H]^−^), found 249.1515; Note: Due to the rapid oxidation of the aldehyde upon standing only the carboxylic acid was identified during HRMS analysis.

#### (*E*)-(((2-Methyl-8-nitrooct-7-en-1-yl)oxy)methyl)benzene (**10**)

To a solution of freshly prepared **18** (931 mg, 3.97 mmol, 1.0 equiv) and bromonitromethane (277 μL, 3.97 mmol, 1.0 equiv) in THF (39.7 mL) was added sodium iodide (599 mg, 3.97 mmol, 1.0 equiv), and the mixture stirred for 2 h at room temperature. Upon completion, the reaction was quenched with 1 N HCl and extracted with ethyl acetate (×3). Combined organics were washed with saturated sodium thiosulfate, dried over sodium sulfate, filtered, and made concentrated. The resulting orange oil was then dissolved in THF (18 mL) and sparged with N_2_ for 3 min before cooling to −78 °C. Samarium(II) iodide (0.1 M in THF, 70.5 mL, 7.05 mmol, 1.8 equiv) was added dropwise, then the solution was warmed to -40 °C and stirred for 4 h (Monitoring by NMR, quench aliquot with 1 N HCl, then extract with dichloromethane). The reaction was diluted in ethyl acetate, quenched with 0.1 N HCl, and brought to room temperature. The layers were separated, and the aqueous portion was extracted with ethyl acetate (×2). The combined organics was washed with sodium thiosulfate, dried over anhydrous Na_2_SO_4_, filtered, and made concentrated. Crude product was purified using a Teledyne ISCO Combi-Flash system (liquid loading, 40G column, 0–30% EtOAc, 20 min run) to give 1**9** (594 mg, 2.14 mmol, 54% yield) as a clear oil.

^1^H NMR (400 MHz CDCl_3_) δ 7.16–7.29 (m, 6H), 6.89 (dt, *J* = 1.4, 13.4 Hz, 1H), 4.42 (s, 2H), 3.17–3.25 (m, 2H), 2.21 (qd, *J* = 1.4, 7.2 Hz, 2H), 1.68 (o, J = 6.5 Hz, 1H), 1.18–1.47 (m, 5H), 1.02–1.11 (m, 1H), 0.85 (d, *J* = 6.7 Hz, 3H); ^13^C{^1^H} NMR (101 MHz CDCl_3_) δ142.8, 139.7, 138.8, 128.5, 127.7, 127.6, 75.8, 73.1, 33.5, 33.4, 28.5, 28.1, 26.6, 17.2; HRMS (ESI) calculated for C_16_H_23_NO_3_ 295.2016 ([M + NH_4_]^+^), found 295.2013.

#### Methyl 4-(6-Methoxy-3,4-dihydronaphthalene-1(2*H*)-ylidene)but-2-enoate (**21**)

Compound was prepared using a previous method with modifications.^19^ To an oven-dried three-necked-flask equipped with a reflux condenser and stir bar was added zinc–copper couple (27.07 g, 209.98 mmol, 3.7 equiv) and methyl (*E*/*Z*)-methyl 4-bromocrotonate (565 μL, 4.81 mmol, 8 mol %) in benzene (60 mL). The suspension was then warmed to 50 °C before the addition of diisobutylaluminum hydride (1.0 M in THF, 4.8 mL, 5.68 mmol, 10 mol %) and stirred for 1 h. The solution was then gently heated to 80 °C before the slow addition of (*E*/*Z*)-methyl 4-bromocrotonate (10 mL, 85.13 mmol, 1.5 equiv) and 7-methoxy-4-tetralone (10 g, 56.75 mmol, 1.0 equiv) in benzene (40 mL). Upon full addition, the solution was brought to reflux at 90 °C with vigorous stirring. After 1 h, additional (*E*/*Z*)-methyl 4-bromocrotonate (10 mL, 85.13 mmol, 1.5 equiv) was added over the course of an hour in four equal portions, and the mixture was stirred at 90 °C for an additional 2 h. At this point, the reaction was filtered over a pad of Celite and washed with excess ethyl acetate. Organics were dried over anhydrous Na_2_SO_4_, filtered, and made concentrated. Crude product was purified twice using a Teledyne ISCO Combi-Flash system (solid loading, 220G column, 0–30% EtOAc, 35 min run) to give **21** (5.42 g, 20.98 mmol, 36% yield) as a yellow solid.

^1^H NMR (400 MHz CDCl_3_) δ 7.78 (dd, *J* = 11.8, 15.0 Hz, 1H), 7.63 (d, *J* = 8.9 Hz, 1H), 6.76 (dd, *J* = 2.7, 8.8 Hz, 1H), 6.64–6.66 (m, 2H), 5.93 (d, *J* = 15.0 Hz, 1H), 3.81 (s, 3H), 3.76 (s, 3H), 2.75–2.82 (m, 4H), 1.87 (quint, *J* = 6.3 Hz, 2H); HRMS (ESI) calculated for C_16_H_18_O_3_ 259.1329 ([M + H]^+^), found 259.1331.

#### Methyl 4-(6-Methoxynaphthalen-1-yl)butanoate (**22**)

In a round-bottom flask, **21** (5.42 g, 20.98 mmol, 1.0 equiv) was dissolved in ethylene glycol (109 mL) followed by the addition of potassium hydroxide (12.03 g, 210.67 mmol, 10.0 equiv). The reaction was then heated to 180 °C and stirred overnight. The following morning, the reaction was diluted with water, extracted with hexanes, made acidic with 4 N HCl (pH ∼ 1), and extracted with ethyl acetate (×3). Combined organics were washed with brine, dried over Na_2_SO_4_, filtered, and made concentrated. The resulting orange solid was then transferred to a 500 mL round-bottom flask with potassium carbonate (8.7 g, 62.02 mmol, 3.0 equiv), which were suspended in dimethylformamide (69 mL) followed by the addition of iodomethane (3.9 mL, 62.02 mmol, 3.0 equiv). The reaction was then stirred for 2 h at room temperature, after which an additional of 1.3 mL (20.67 mmol, 1.0 equiv) of iodomethane was added, and the mixture was stirred for another 2 h, by which point LCMS indicated full conversion. The reaction was diluted in water and extracted with ethyl acetate (×3). Combined organics were then washed with water and then brine, dried over anhydrous Na_2_SO_4_, filtered, and made concentrated. **22** (4.7 g, 18.20 mmol, 87% yield) was obtained as an orange-brown oil that was used without further purification.

^1^H NMR (400 MHz CDCl_3_) δ 7.97 (d, *J* = 9.1 Hz, 1H), 7.62 (d, *J* = 8.2 Hz, 1H), 7.4 (t, 1H), 7.15–7.20 (m, 3H), 3.93 (s, 3H), 3.69 (s, 3H), 3.08 (t, *J* = 7.7 Hz, 2H), 2.42 (t, *J* = 7.3 Hz, 2H), 2.08 (quint, *J* = 7.5 Hz, 2H); ^13^C{^1^H} NMR (101 MHz CDCl_3_) δ 174.1, 157.4, 137.7, 135.3, 127.4, 126.3, 125.8, 125.5, 124.2, 118.6, 106.8, 55.4, 51.6, 33.8, 32.5, 26.0; HRMS (ESI) calculated for C_16_H_18_O_3_ 237.1849 ([M + H]^+^), found 259.1329.

#### 4-(6-Methoxynaphthalen-1-yl)butanoic Acid (**23**)

In a round-bottom flask, **22** (4.7 g, 18.2 mmol, 1.0 equiv) was dissolved in a mixture of THF (72.8 mL) and water (18.2 mL), followed by the addition of sodium hydroxide (1.49 g, 36.39 mmol, 2.0 equiv). The reaction was then heated to 80 °C and stirred for 2 h. Upon completion, the reaction was made acidic with 4 N HCl (pH ∼ 1) and extracted with ethyl acetate (×3). Combined organics were dried over anhydrous Na_2_SO_4_, filtered, and made concentrated. **23** (4.27 g, 17.48 mmol, 96% yield) was obtained as a forest green solid.

^1^H NMR (400 MHz DMSO-*d*_6_) δ 8.02 (d, *J* = 9.2 Hz, 1H), 7.67 (d, *J* = 8.2 Hz, 1H), 7.36 (dd, *J* = 7.2, 8.1 Hz, 1H), 7.32 (d, *J* = 2.6 Hz, 1H), 7.16–7.19 (m, 2H), 3.87 (s, 3H), 3.00 (t, *J* = 7.8 Hz, 2H), 2.31 (t, *J* = 7.2 Hz, 2H), 1.87 (quint, *J* = 7.5 Hz, 2H); ^13^C{^1^H} NMR (101 MHz DMSO-*d*_6_) δ 174.4, 156.9, 137.8, 134.9, 126.8, 126.2, 125.5, 125.4, 123.8, 118.3, 106.8, 55.2, 33.3, 31.7, 26.0; HRMS (ESI) calculated for C_15_H_16_O_3_ 245.1172 ([M + H]^+^), found 245.1174. Note: carboxylic acid OH is not observed in ^1^H NMR

#### 7-Methoxy-3,4-dihydrophenanthren-1(2*H*)-one (**24**)

In a vial, **23** (1.16 g, 4.75 mmol, 1.0 equiv) was dissolved in Eaton’s reagent (5 mL, 31.51 mmol, 6.6 equiv), heated to 100 °C, and stirred for 1 h. Upon full conversion, the material was cooled to 0 °C, and water was slowly added (∼50 mL). The mixture was extracted with ethyl acetate (×3). Combined organics were then washed with brine, dried over anhydrous Na_2_SO_4_, filtered, and made concentrated. **24** (1.06 g, 4.67 mmol, 98% yield) was obtained as a brown solid that was not purified further.

^1^H NMR (400 MHz CDCl_3_) δ 8.10 (d, *J* = 8.7 Hz, 1H), 8.05 (d, *J* = 9.2 Hz, 1H), 7.65 (d, *J* = 8.7 Hz, 1H), 7.24 (dd, *J* = 2.6, 9.2 Hz, 1H), 7.17 (d, *J* = 2.6 Hz, 1H), 3.97 (s, 3H), 3.35 (t, *J* = 6.2 Hz, 2H), 2.74 (t, *J* = 6.6 Hz, 2H), 2.30 (quint, J = 6.4 Hz, 2H); ^13^C{^1^H} NMR (101 MHz CDCl_3_) δ 198.5, 159.6, 143.1, 137.7, 128.5, 126.7, 126.5, 125.9, 123.7, 119.2, 107.0, 55.5, 38.4, 25.8, 22.9; HRMS (ESI) calculated for C_15_H_14_O_2_ 227.1067 ([M + H]^+^), found 227.1066.

#### 7-Methoxy-2-methyl-3,4-dihydrophenanthren-1(2*H*)-one (**9**)

To an oven-dried 100 mL round-bottom flask was added THF (10 mL) and diisopropylamine (0.71 mL, 5.09 mmol, 1.5 equiv). The solution was then cooled to −78 °C before the addition of *n*-butyllithium (2.5 M in hexanes, 1.76 mL, 4.41 mmol, 1.3 equiv). The mixture was then warmed up to 0 °C and stirred for 15 min before cooling back down to −78 °C and the addition of **24** (768 mg, 3.39 mmol, 1.0 equiv) in THF (3 mL). The reaction was then stirred for 2 h at this temperature before the addition of iodomethane (0.27 mL, 4.41 mmol, 1.3 equiv). The reaction was then allowed to gradually warm to room temperature overnight. The following morning, the mixture was quenched with saturated ammonium chloride and extracted with ethyl acetate (×3). Combined organics were then washed with brine, dried over anhydrous Na_2_SO_4_, filtered, and made concentrated. Crude product was purified using a Teledyne ISCO Combi-Flash system (solid loading, 40G column, 0–10% EtOAc in Hexanes, 30 min run) was obtained as **9** (218 mg, 0.91 mmol, 26% yield) an off-white solid.

^1^H NMR (400 MHz CDCl_3_) δ 8.08 (d, *J* = 8.7 Hz, 1H), 8.00 (d, *J* = 9.2 Hz, 1H), 7.62 (d, *J* = 8.7 Hz, 1H), 7.21 (dd, *J* = 2.6, 9.2 Hz, 1H), 7.14 (d, *J* = 2.6 Hz, 1H), 3.94 (s, 3H), 3.48 (dt, *J* = 4.4, 17.3, 1H), 3.18–3.26 (m, 1H), 2.62–2.70 (m, 1H), 2.32–2.39 (m, 1H), 1.92–2.04 (m, 1H), 1.31 (d, *J* = 6.8, 3H); ^13^C{^1^H} NMR (101 MHz CDCl_3_) δ 200.9, 159.5, 142.5, 137.5, 128.2, 126.5, 126.5, 125.9, 124.0, 119.1, 107.0, 55.5, 41.6, 30.9, 25.2, 15.5; HRMS (ESI) calculated for C_16_H_16_O_2_ 241.1223 ([M + H]^+^), found 241.1223.

#### 2-(8-(Benzyloxy)-7-methyl-1-nitrooctan-2-yl)-7-methoxy-2-methyl-3,4-dihydrophenanthren-1(2*H*)-one-7-methoxy-2-methyl-3,4-dihydrophenanthren-1(2*H*)-one-(*E*)-(((2-methyl-9-nitronon-8-en-1-yl)oxy)methyl)benzene (1/1/1) (**25**)

In an oven-dried vial, **9** (87 mg, 0.36 mmol, 1.0 equiv) was dissolved in THF (1.8 mL) and cooled to −78 °C, followed by the addition of sodium bis(trimethylsilyl)amide (1.0 M in THF, 500 μL, 0.5 mmol, 1.4 equiv). The reaction was then warmed to −45 °C before the addition of **10** (100 mg, 0.36 mmol, 1.0 equiv) in THF (1.8 mL). The reaction was then stirred for 30 min at which point both LCMS and TLC indicated consumption of the nitroalkene. Saturated ammonium chloride was added to quench the reaction, and the mixture was extracted with ethyl acetate (×3). Organics were filtered through a phase separator and made concentrated. Crude product was purified using a Teledyne ISCO Combi-Flash system (liquid loading, 24G column, 0–10% EtOAc in Hexanes, 20 min run, starting material elution ∼2% and product elution ∼9%). **25** (93 mg, 0.18 mmol, 50% yield) as a complex mixture of diastereomers and as a clear syrup.

^1^H NMR (400 MHz, CDCl_3_) δ 8.06 (d, *J* = 8.7 Hz, 1H), 8.02 (d, *J* = 9.5 Hz, 1H), 7.66 (d, *J* = 8.7 Hz, 1H), 7.36–7.28 (m, 5H), 7.28–7.21 (m, 3H), 7.16 (d, *J* = 2.6 Hz, 1H), 4.55 (ddd, *J* = 13.3, 4.8, 2.0 Hz, 1H), 4.48 (s, 2H), 4.29 (ddd, *J* = 13.4, 6.5, 1.6 Hz, 1H), 3.96 (s, 3H), 3.38–3.20 (m, 4H), 2.95–2.88 (m, 1H), 2.04–1.98 (m, 1H), 1.79–1.67 (m, 1H), 1.61–1.29 (m, 5H), 1.29–1.24 (m, 2H), 1.20 (s, 3H), 0.90 (dd, *J* = 6.7, 1.2 Hz, 3H).; ^13^C NMR (101 MHz, CDCl_3_) δ 201.04, 159.84, 141.14, 138.94, 137.72, 128.44, 127.66, 127.54, 127.01, 126.50, 126.44, 126.20, 124.60, 119.39, 107.10, 76.00, 73.09, 60.53, 55.59, 47.02, 40.94, 33.52, 30.50, 30.24, 28.45, 27.31, 21.83, 19.43, 17.23, 14.34; HRMS (ESI) calculated for C_32_H_39_NO_5_ 517.2828 ([M + H]^+^), found 518.2897.

#### 1-(6-Hydroxy-5-methylhexyl)-11a-methyl-2,10,11,11a-tetrahydro-1*H*-naphtho[1,2-*g*]indol-7-ol-1-(6-(benzyloxy)-5-methylhexyl)-7-methoxy-11a-methyl-2,10,11,11*a*-tetrahydro-1*H*-naphtho[1,2-*g*]indole (1/1) (**26**)

In a round-bottom flask, **25** (600 mg, 1.16 mmol, 10 equiv) was dissolved in ethanol (11.6 mL), followed by the addition of one spatula full of Raney nickel (suspension in water). The mixture was then degassed and placed under an atmosphere of nitrogen, then an atmosphere of hydrogen gas and stirred overnight at room temperature with rigorous stirring. Upon completion, the reaction was degassed and placed under an atmosphere of nitrogen before filtering through a pad of Celite. The filtrate was then made concentrated and purified using a Teledyne ISCO Combi-Flash system (liquid loading, 40G column, 0–60% EtOAc in Hexanes, 25 min run) to give **26** (189 mg, 0.40 mmol, 35% yield) as a mixture of diastereomers and a white wax.

^1^H NMR (400 MHz CDCl_3_) δ 8.12 (d, *J* = 8.7 Hz, 1H), 7.98 (d, *J* = 9.2 Hz, 1H), 7.64 (d, *J* = 8.7 Hz, 1H), 7.28–7.36 (m, 5H), 7.20 (dd, *J* = 2.6 Hz, *J* = 9.2 Hz, 1H), 7.15 (d, *J* = 2.6 Hz, 1H), 4.52 (s, 2H), 4.17 (dd, *J* = 7.5 Hz, 15.2 Hz, 1H), 3.94 (s, 3H), 3.19–3.50 (m, 5H), 2.24 (dd, *J* = 4.8 Hz, *J* = 13.0 Hz, 1H), 2.03–2.07 (m, 1H), 1.77–1.87 (m, 2H), 1.31–1.58 (m, 7H), 1.14–1.21 (m, 1H), 0.95–0.96 (m, 6H^13^C NMR) (101 MHz, CDCl_3_) δ 179.15, 158.47, 138.95, 136.47, 136.24, 128.46, 127.66, 127.57, 127.12, 126.04, 125.92, 125.12, 124.02, 118.67, 107.20, 76.11, 73.14, 63.84, 55.49, 50.60, 47.85, 35.17, 33.73, 33.59, 29.21, 27.98, 27.50, 23.48, 17.28, 14.10; HRMS (ESI) calculated for C_32_H_39_NO_2_ 470.3054 ([M + H]^+^), found 470.3055.

#### 1-(6-Hydroxy-5-methylhexyl)-11a-methyl-2,10,11,11a-tetrahydro-1*H*-naphtho[1,2-*g*]indol-7-ol-1-(6-(benzyloxy)-5-methylhexyl)-7-methoxy-11a-methyl-2,10,11,11a-tetrahydro-1*H*-naphtho[1,2-*g*]indole (1/1) (**8**)

A solution of boron tribromide (61 μL, 0.65 mmol, 2.2 equiv) in dichloromethane (1.5 mL) was added via syringe to a stirring solution of **26** (138 mg, 0.29 mmol, 1.0 equiv) in dichloromethane (1.5 mL) at −78 °C. After 15 min, LCMS indicated the loss of the benzyl group, and the solution was then warmed to −40 °C and stirred for an additional 2 h. Upon completion, excess saturated sodium bicarbonate was added, and the aqueous layer was extracted with 3:1 chloroform:isopropanol (×3). Organics were filtered through a phase separator and made concentrated. Crude product was purified using a Teledyne ISCO Combi-Flash system (liquid loading, 24G column, 0–10% MeOH:DCM, 30 min run). Impurities remained, and the crude was dissolved in DMSO (3 mL) and purified using the Gilson (Basic, 30 × 100 mm^2^ column, 25–75% ACN/0.05% aqueous NH_4_OH, 12 min run). Fractions containing the desired product were concentrated to give **8** (29.1 mg, 0.080 mmol, 27% yield) as a mixture of diastereomers and an orange solid.

^1^H NMR (400 MHz DMSO-*d*_6_) δ 7.97 (d, *J* = 9.6 Hz, 1H), 7.92 (d, *J* = 8.6 Hz, 1H), 7.56 (d, *J* = 8.6 Hz, 1H), 7.14–7.16 (m, 2H), 4.01 (dd, *J* = 7.3 15.0 Hz, 1H), 3.08–3.40 (m, 6H), 2.16 (dd, *J* = 4.4, 12.5 Hz, 1H), 1.91–1.93 (m, 1H), 1.68 (td, *J* = 5.4, 18.8 Hz, 1H), 1.34–1.50 (m, 8H), 1.04–1.06 (m, 1H), 0.83–0.85 (m, 6H); ^13^C{^1^H} NMR (101 MHz DMSO-*d*_6_) δ 177.5, 156.4, 136.2, 135.9, 126.1, 125.6, 125.0, 123.7, 123.1, 118.6, 110.0, 66.3, 62.9, 50.1, 47.1, 35.4, 34.4, 32.9, 28.7, 27.3, 27.0, 22.7, 16.9, 13.6; HRMS (ESI) calculated for C_24_H_31_NO_2_ 366.2428 ([M + H]^+^), found 366.2428. Note: phenolic OH is not observed likely due to peak broadening due to proton exchange.

## Supporting Information Available

NMR spectra of all synthesized compounds (^1^H and ^13^C NMR); and analytical chiral SFC chromatogram of compound **8** (PDF)
