# Supplementary material for: Synthesis of a Hydroxy-15-Azasterol
Source: ACS Omega. 2025 Jan 31;10(5):4784–91. doi: 10.1021/acsomega.4c09907 (PMC11822486; doi:10.1021/acsomega.4c09907)
Supplement: Supplementary file 1 — ao4c09907_si_001.pdf [file ao4c09907_si_001.pdf]

Supporting Information

Synthesis of a Hydroxy-15-azasterol

Caleb A. H. Jones,[1,2,*] Bruce J. Melancon,[1,2] Craig W. Lindsley[1,2,3,*]

Affiliation:

[1]Warren Center for Neuroscience Drug Discovery, Vanderbilt University, Nashville, TN 37232, USA
[2]Department of Pharmacology, Vanderbilt University School of Medicine, Nashville, TN 37232, USA
[3]Department of Chemistry, Vanderbilt University, Nashville TN 37232, USA.
*Corresponding authors

Table of Contents

1H and 13C{1H} spectra of compounds: S3

Analytical Chiral SFC Chromatogram of 8 .... S29

1H and 13C{1H} spectra of compounds:

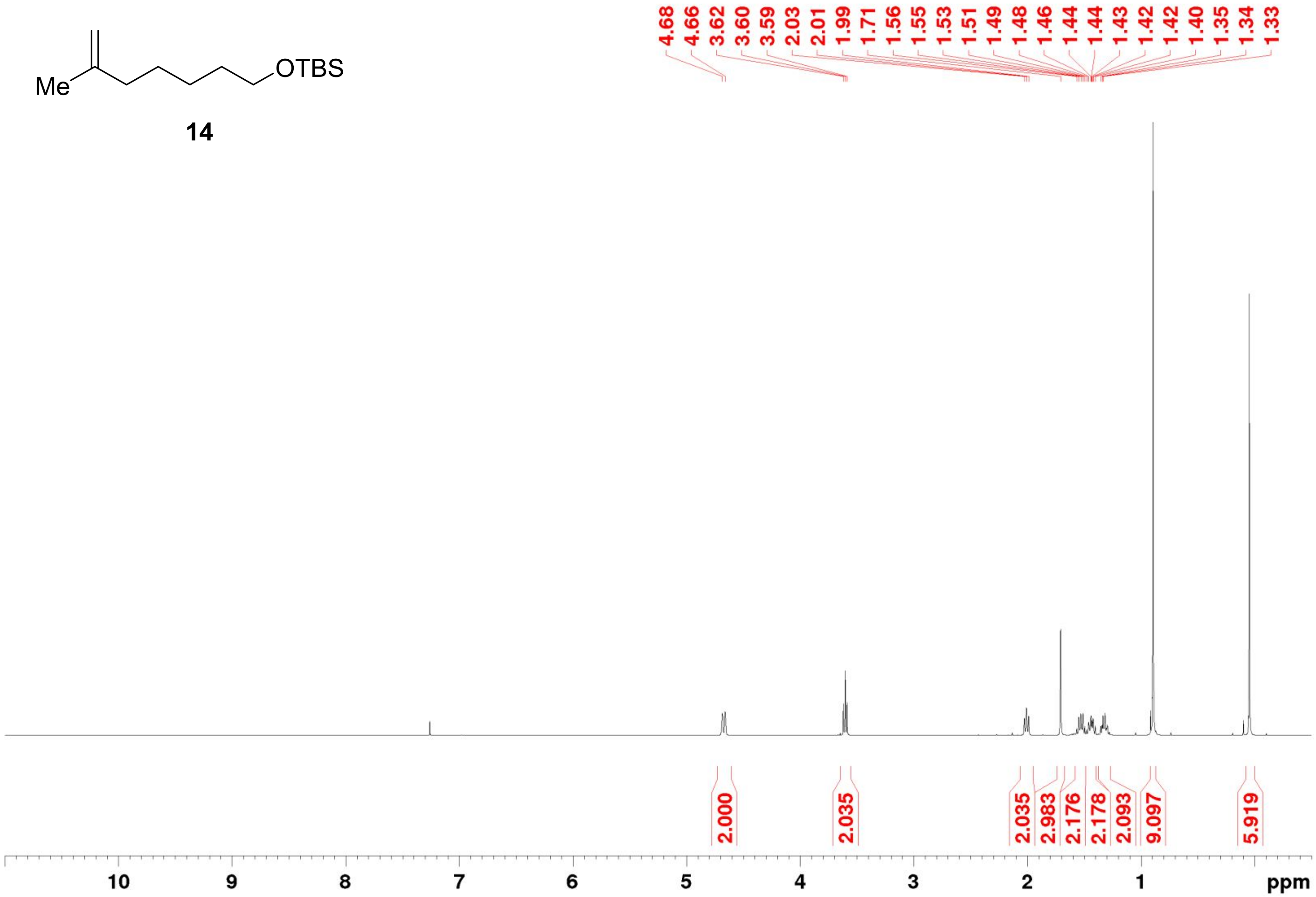

Figure S1. ^{1}H NMR spectrum of **14**

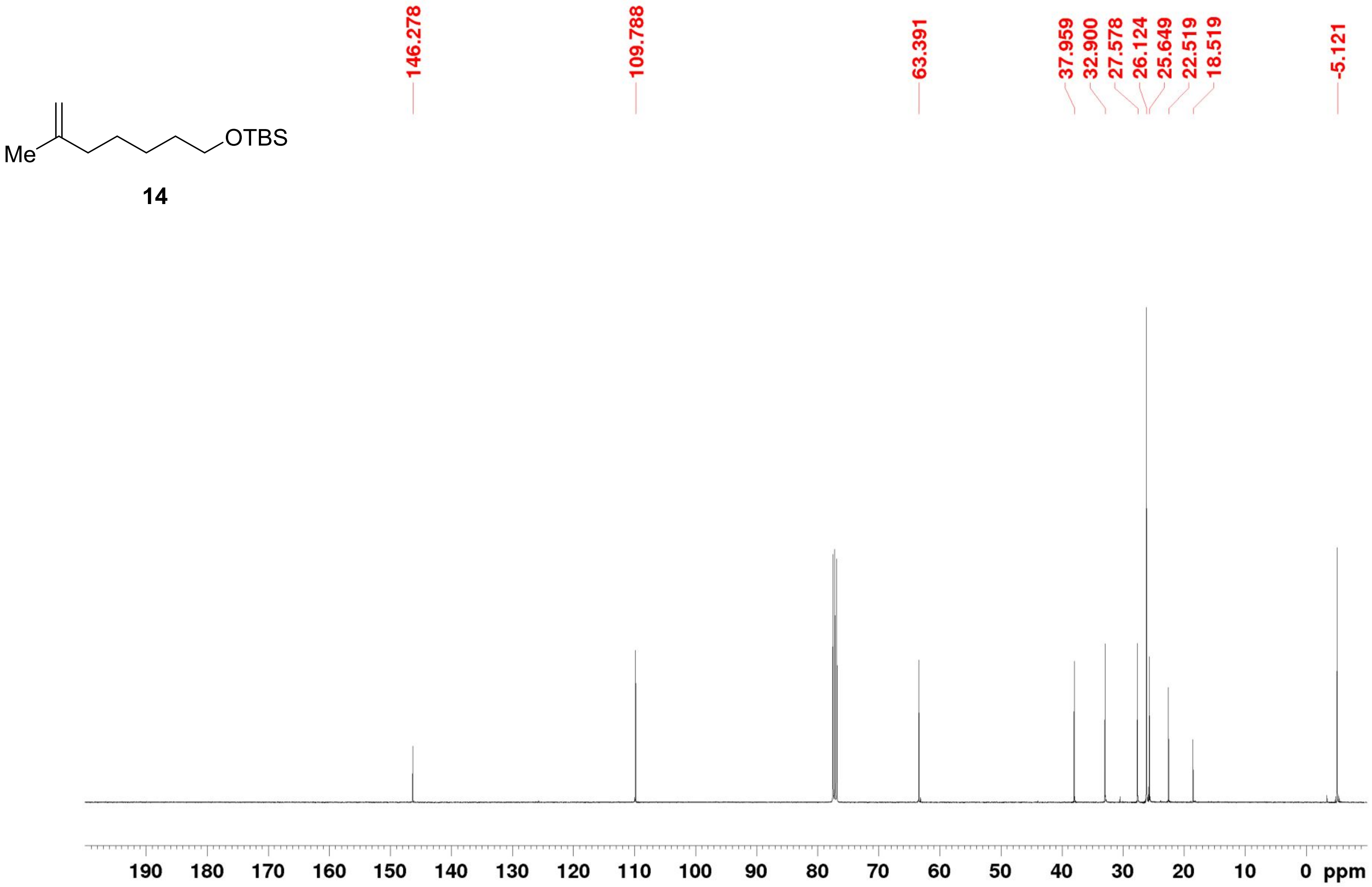

Figure S2. $^{13}C\{^1H\}$ NMR spectrum of **14**

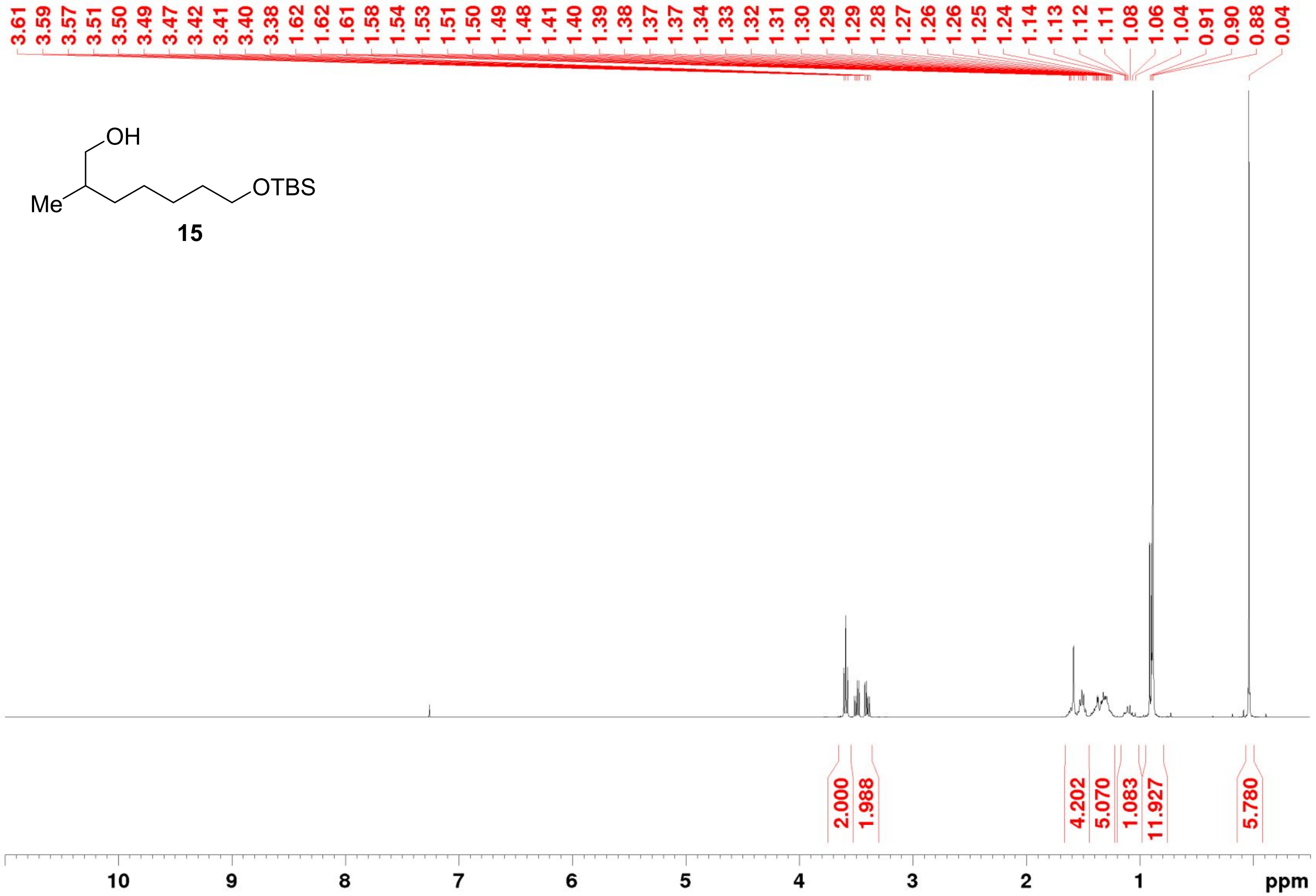

Figure S3. ^{1}H NMR spectrum of 1**5**

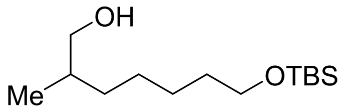

15

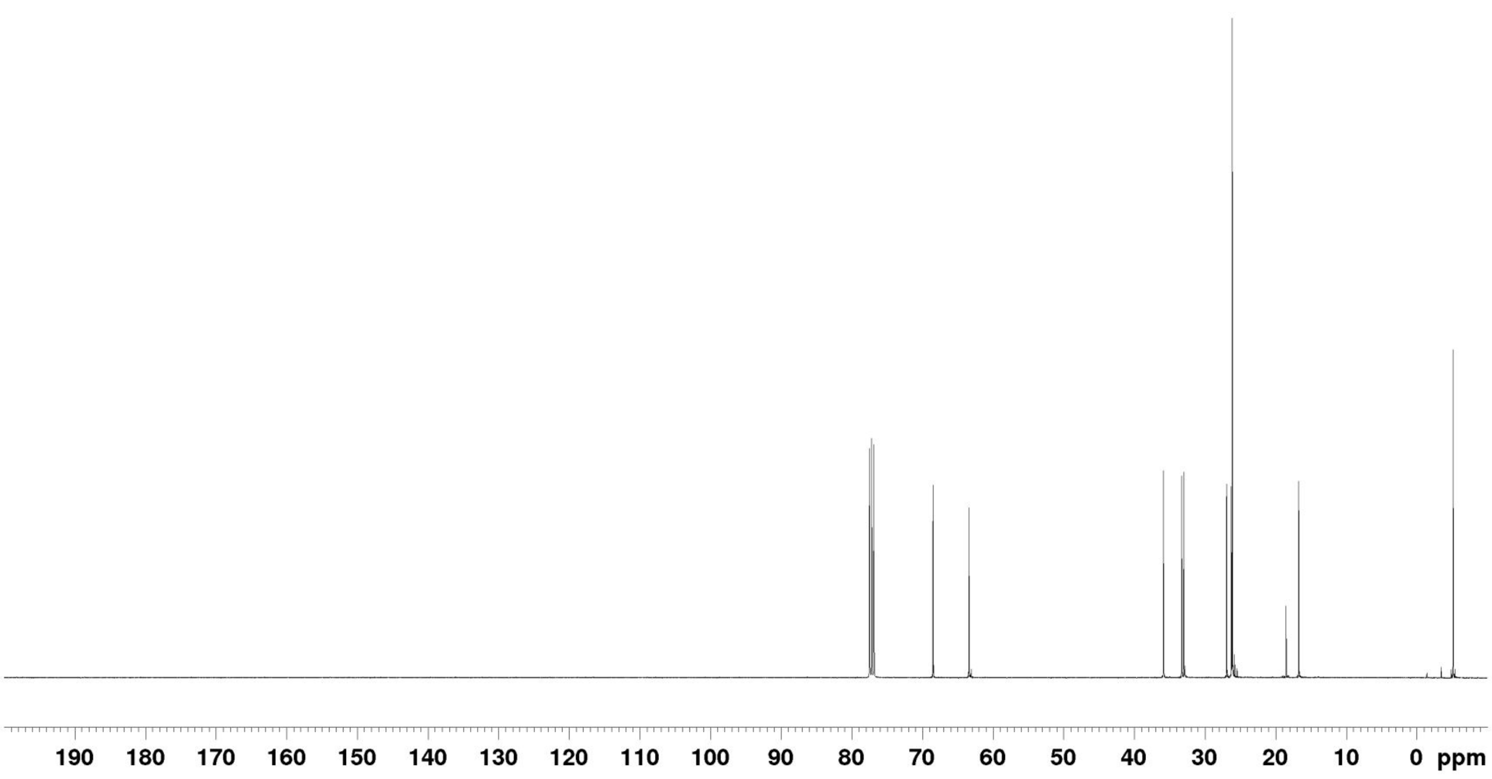

Figure S4. $^{13}C\{^{1}H\}$ NMR spectrum of 1**5**

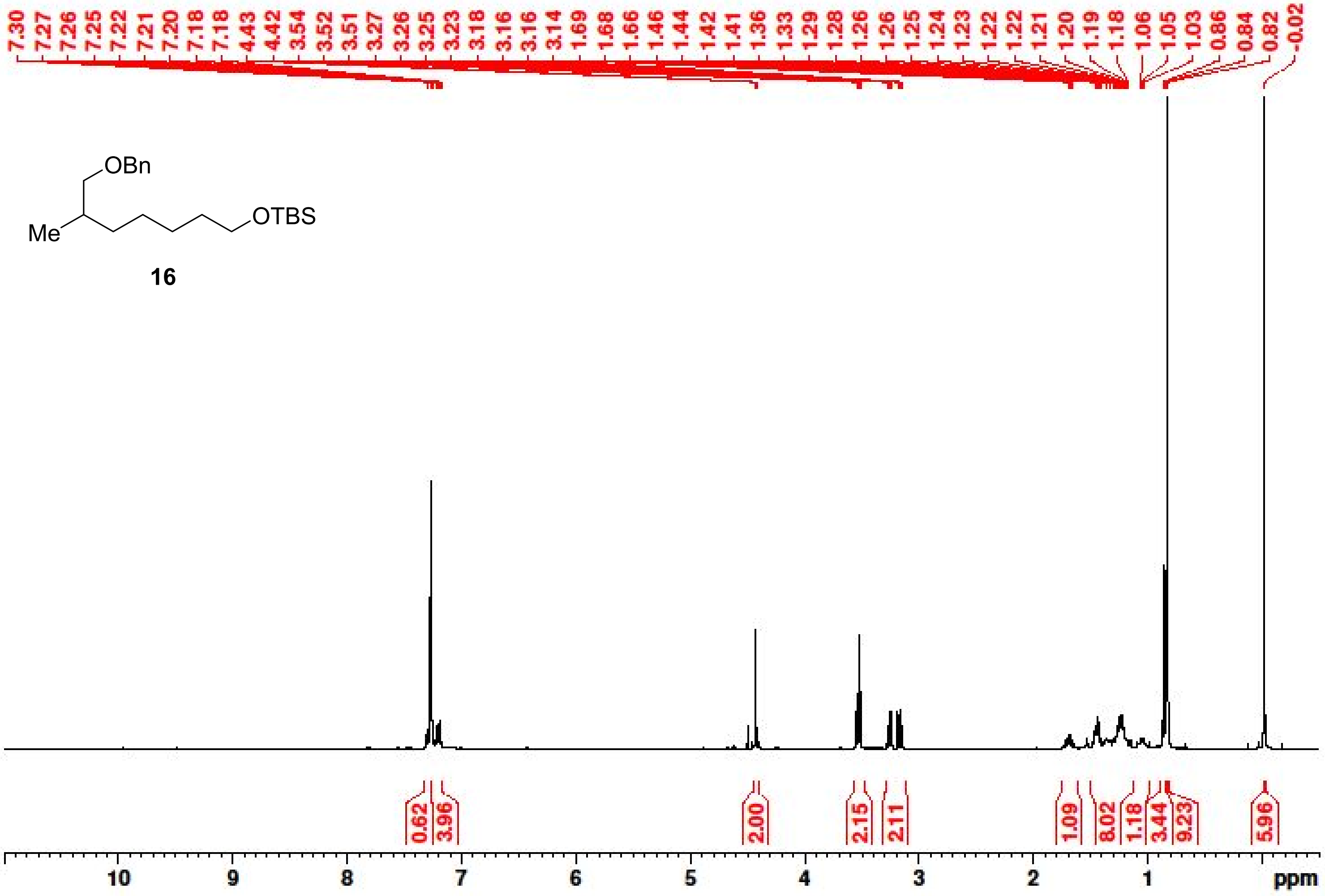

Figure S5. ^{1}H NMR spectrum of 16

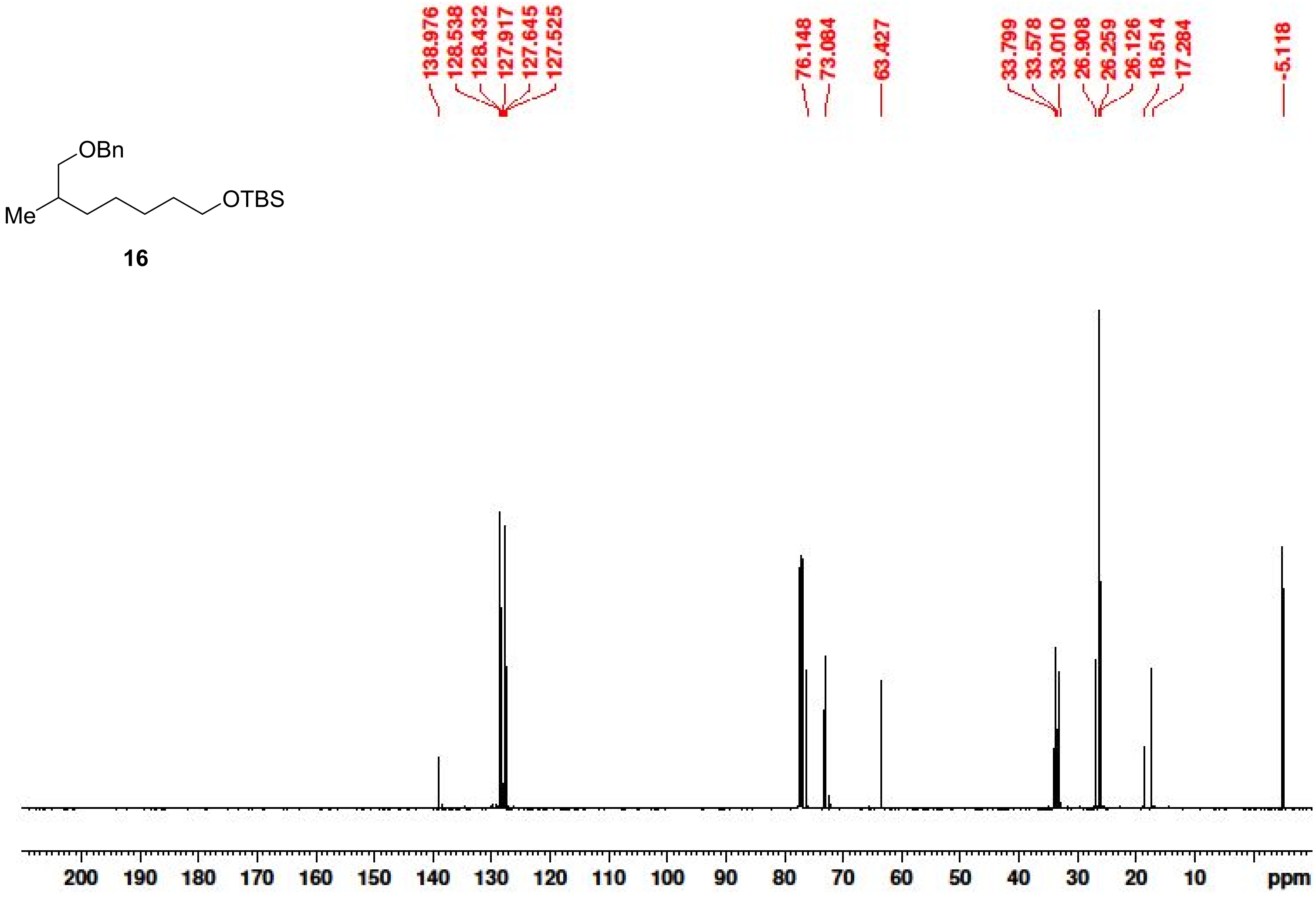

Figure S6. $^{13}C\{^{1}H\}$ NMR spectrum of **16**

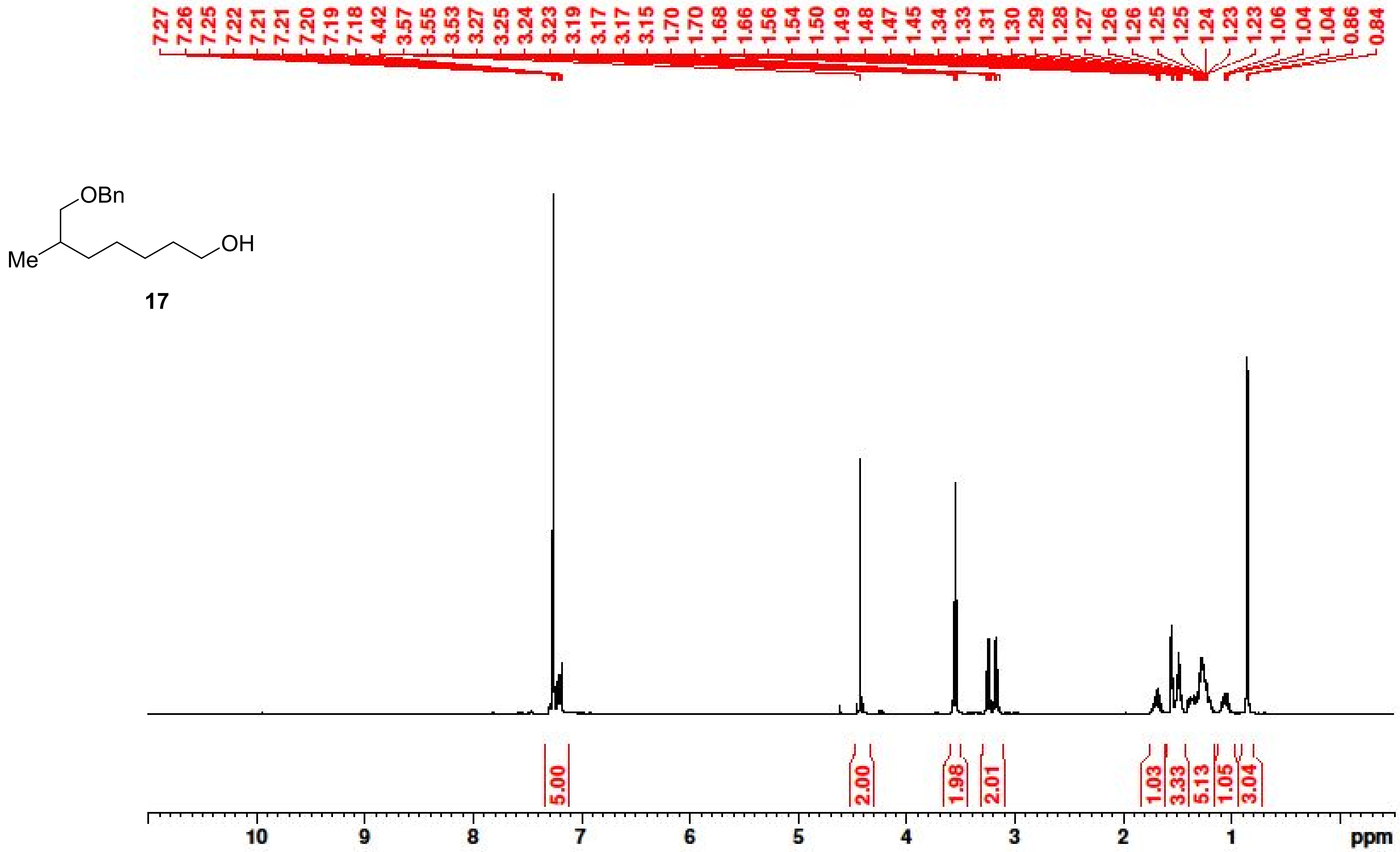

Figure S7. ¹H NMR spectrum of **17**

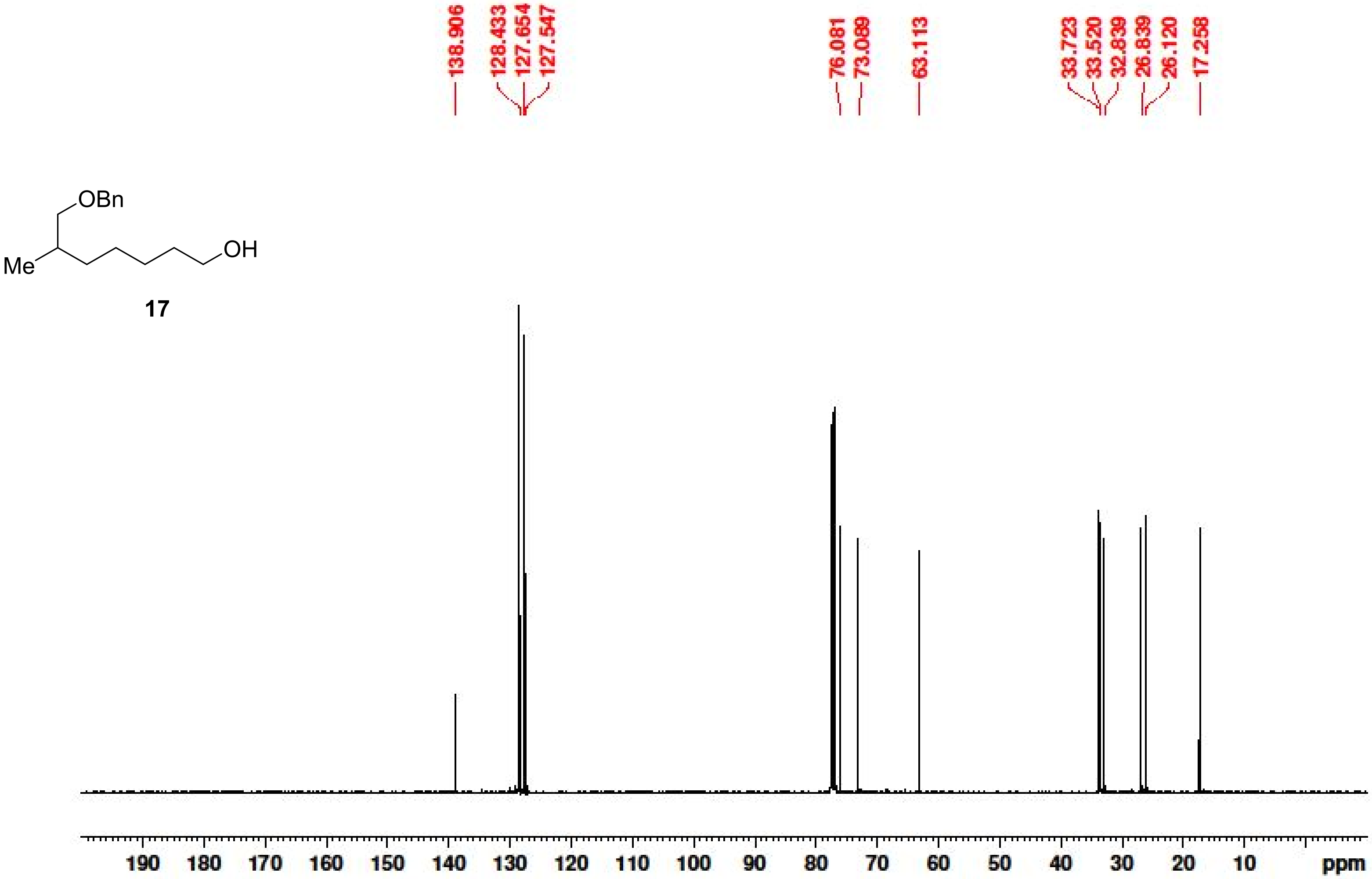

17

Figure S8. $^{13}C\{^{1}H\}$ NMR spectrum of **17**

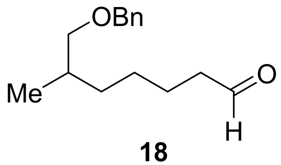

18

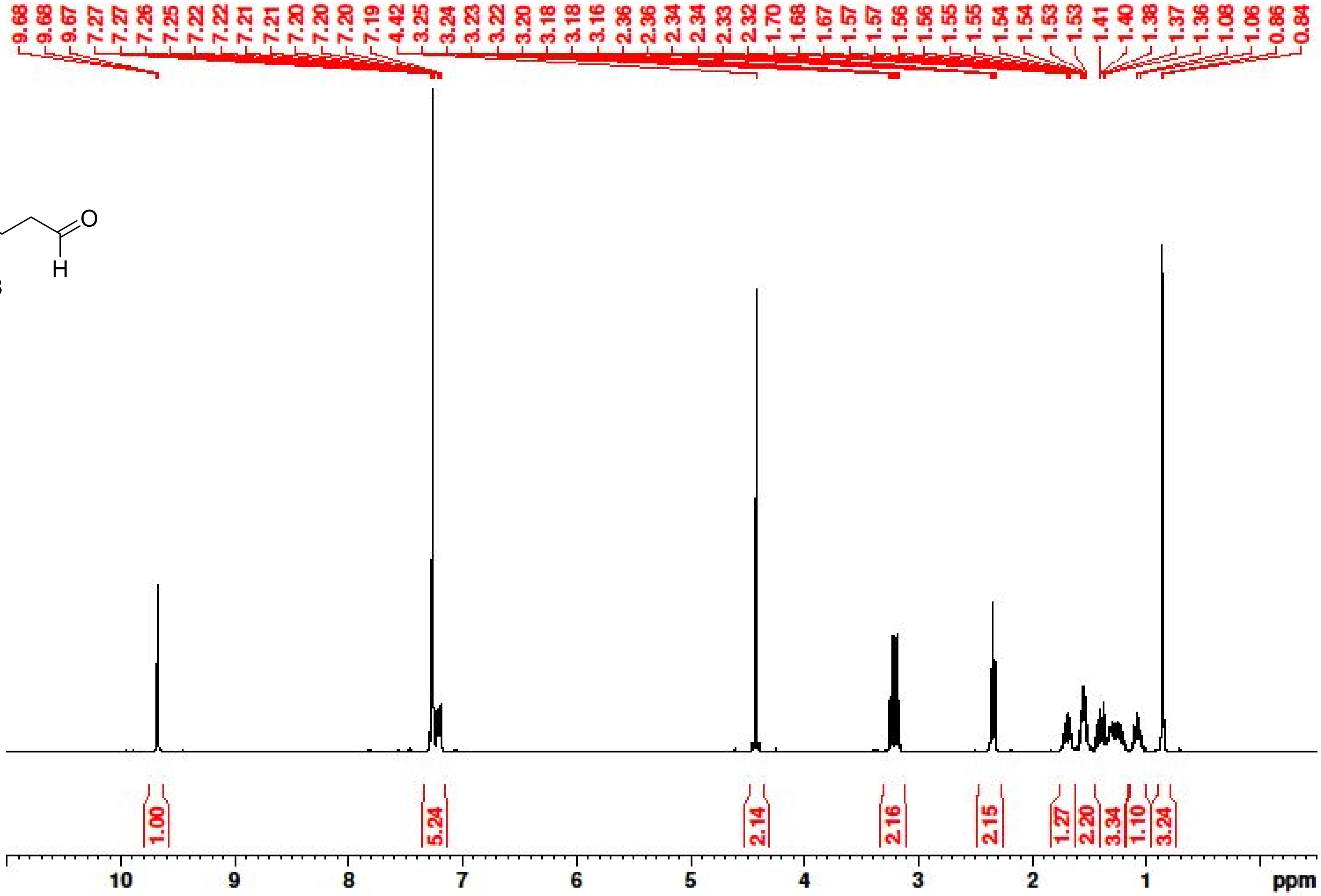

Figure S9. ^{1}H NMR spectrum of **18**

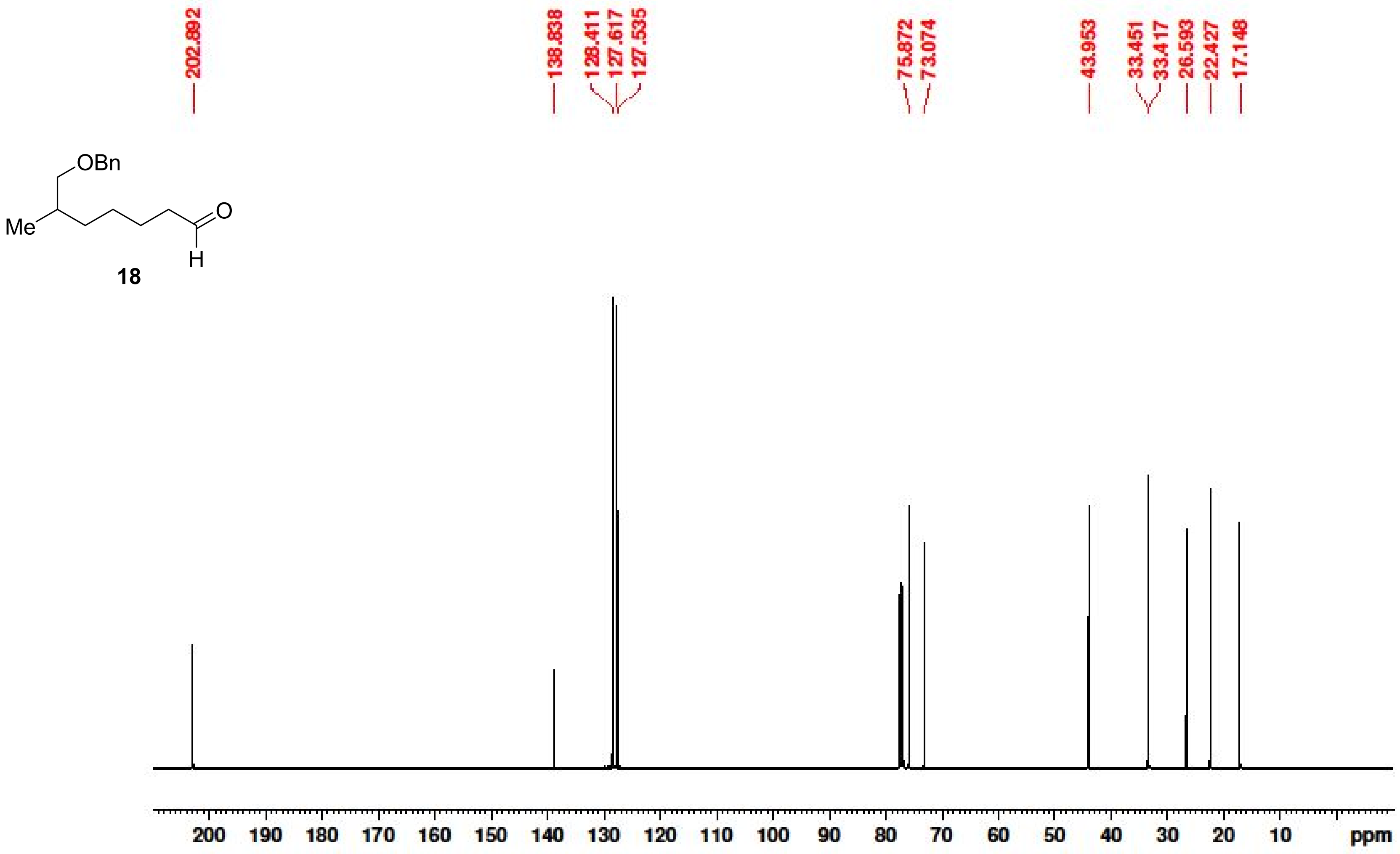

Figure S10. $^{13}C\{^{1}H\}$ NMR spectrum of 18

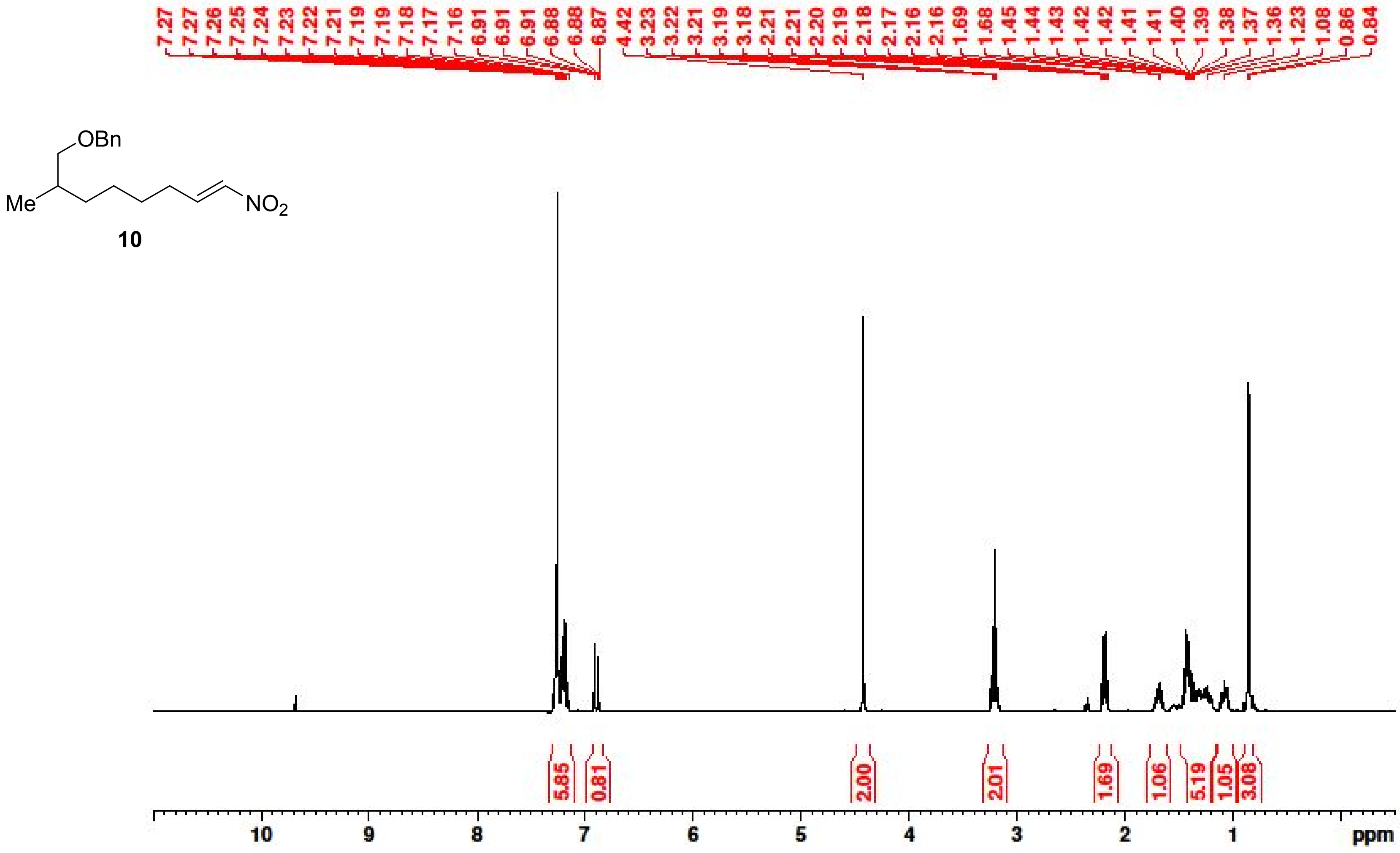

Figure S11. ^{1}H NMR spectrum of **10**

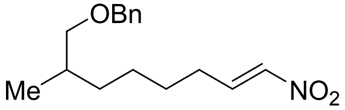

10

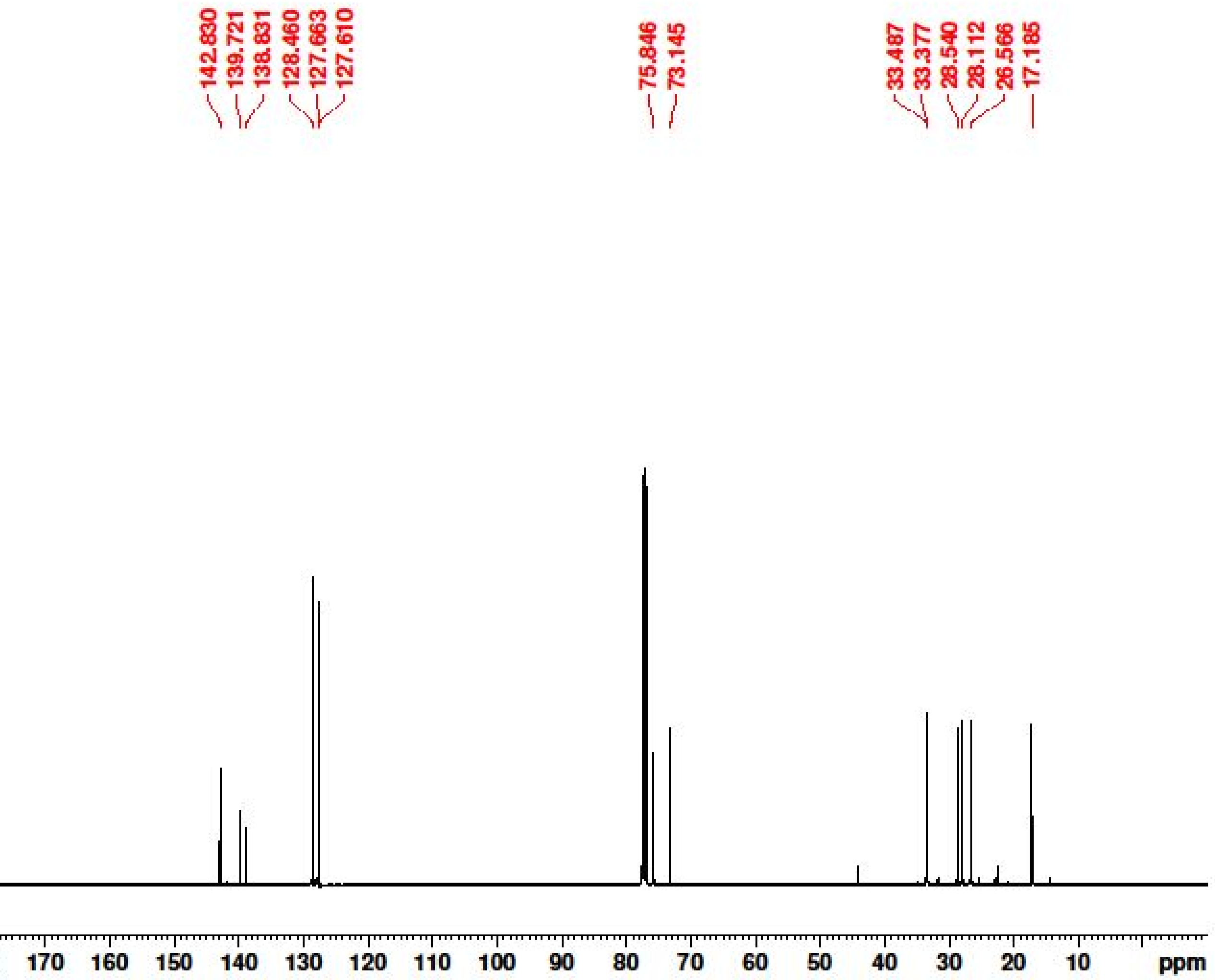

Figure S12. $^{13}C\{^{1}H\}$ NMR spectrum of **10**

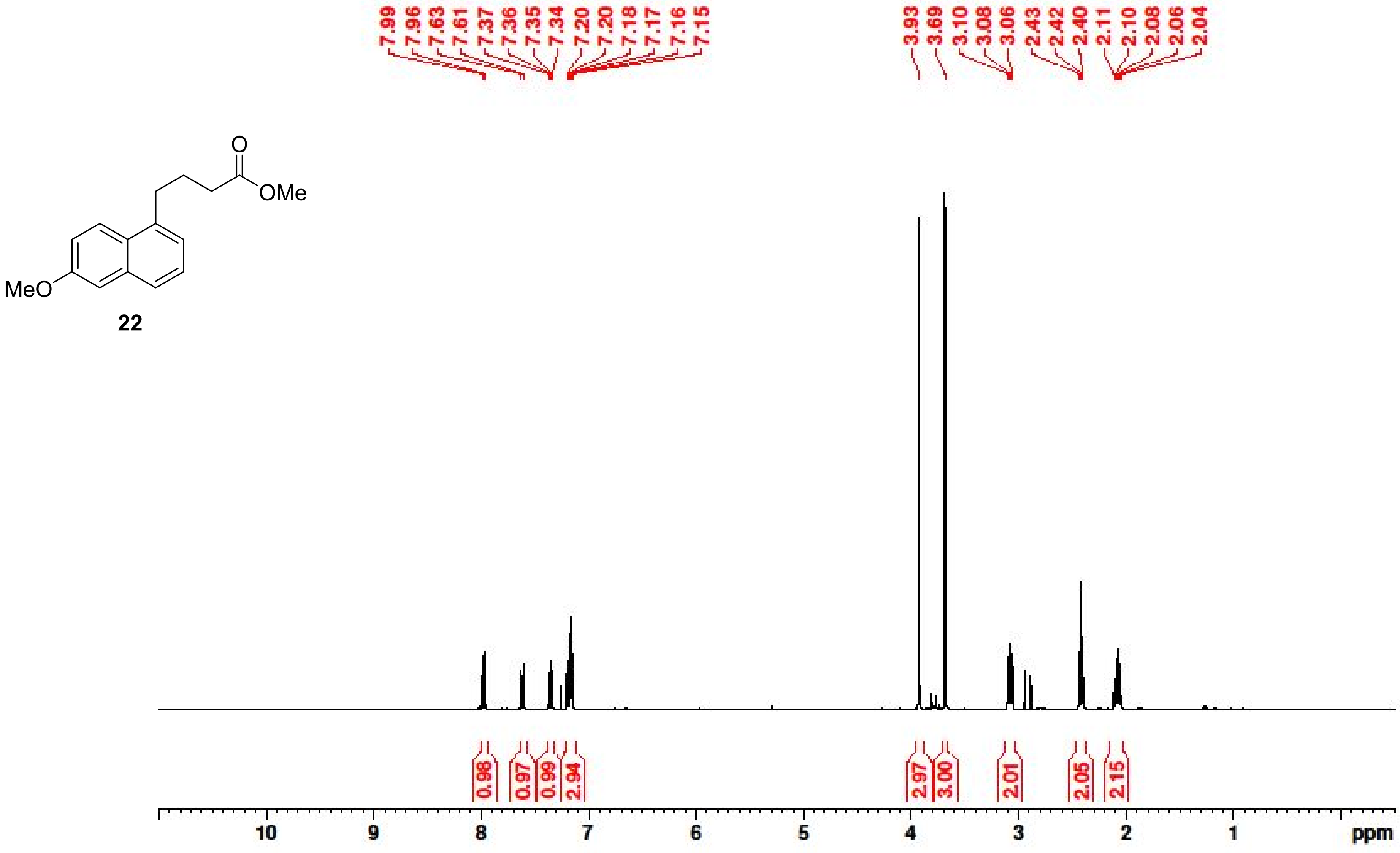

Figure S13. ^{1}H NMR spectrum of **22**

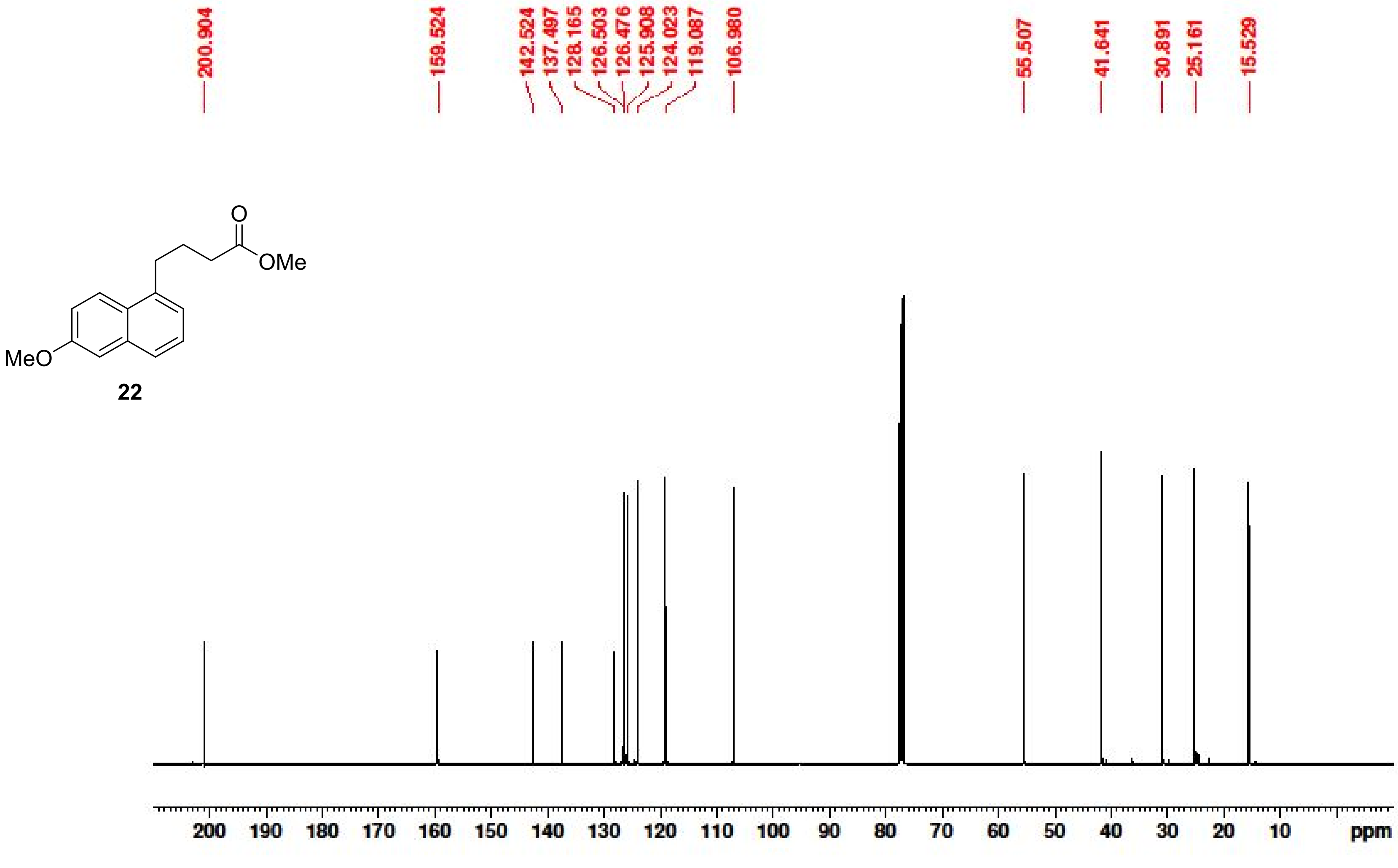

Figure S14. $^{13}C\{^{1}H\}$ NMR spectrum of **22**

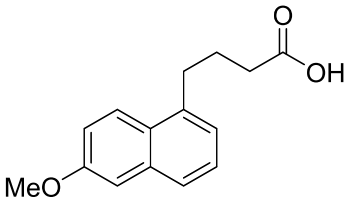

23

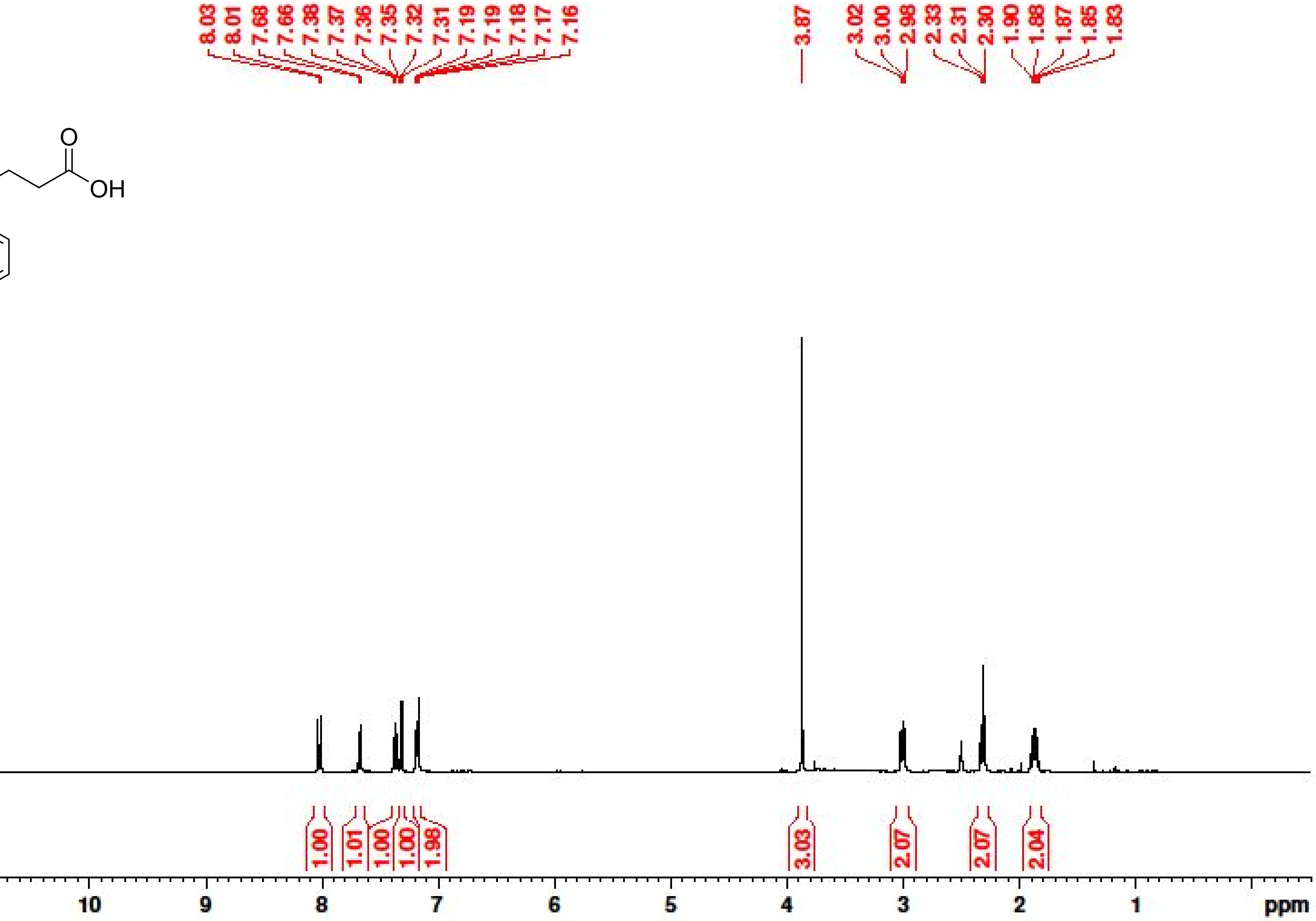

Figure S15. ^{1}H NMR spectrum of **23**

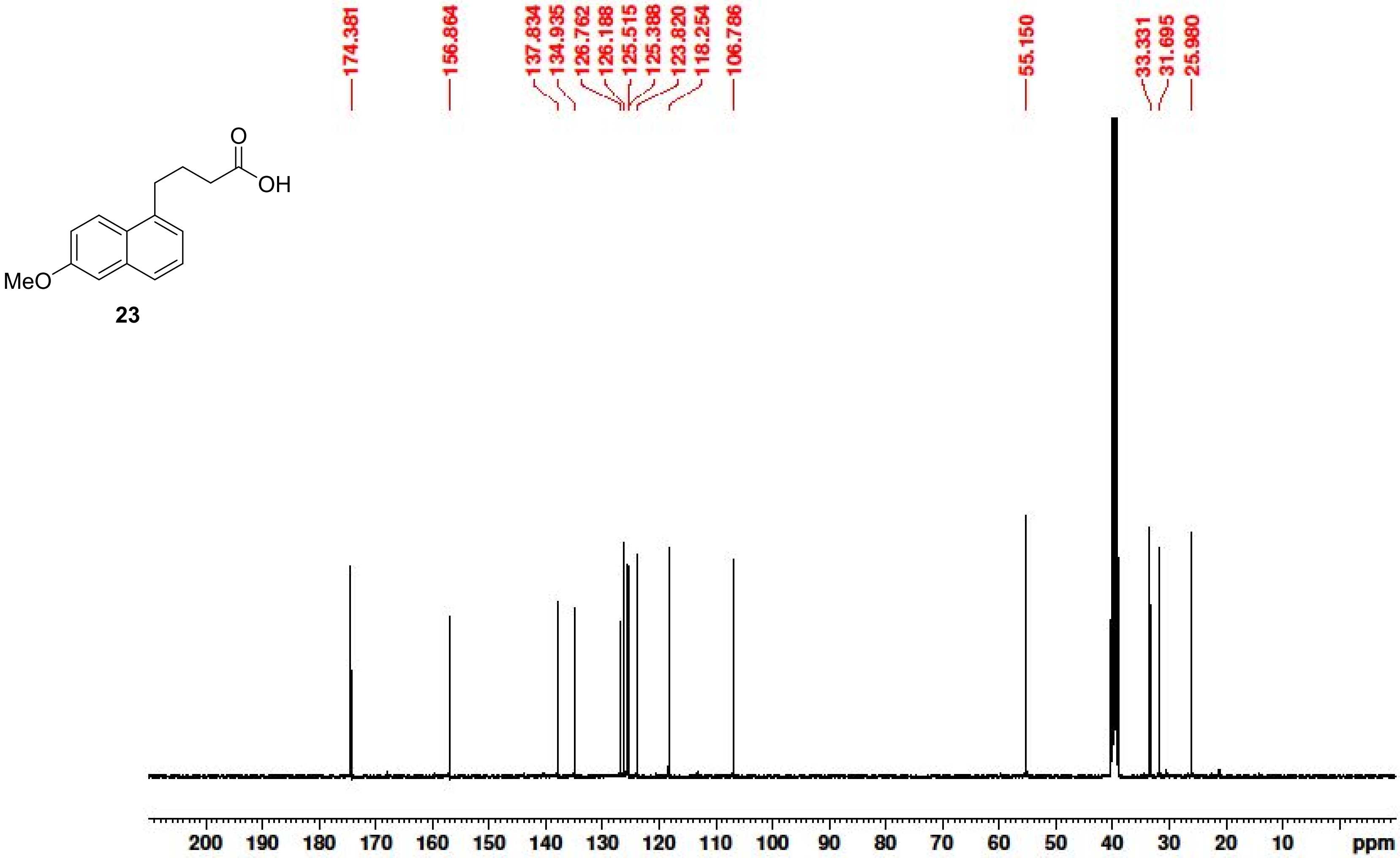

Figure S16. $^{13}C\{^{1}H\}$ NMR spectrum of **23**

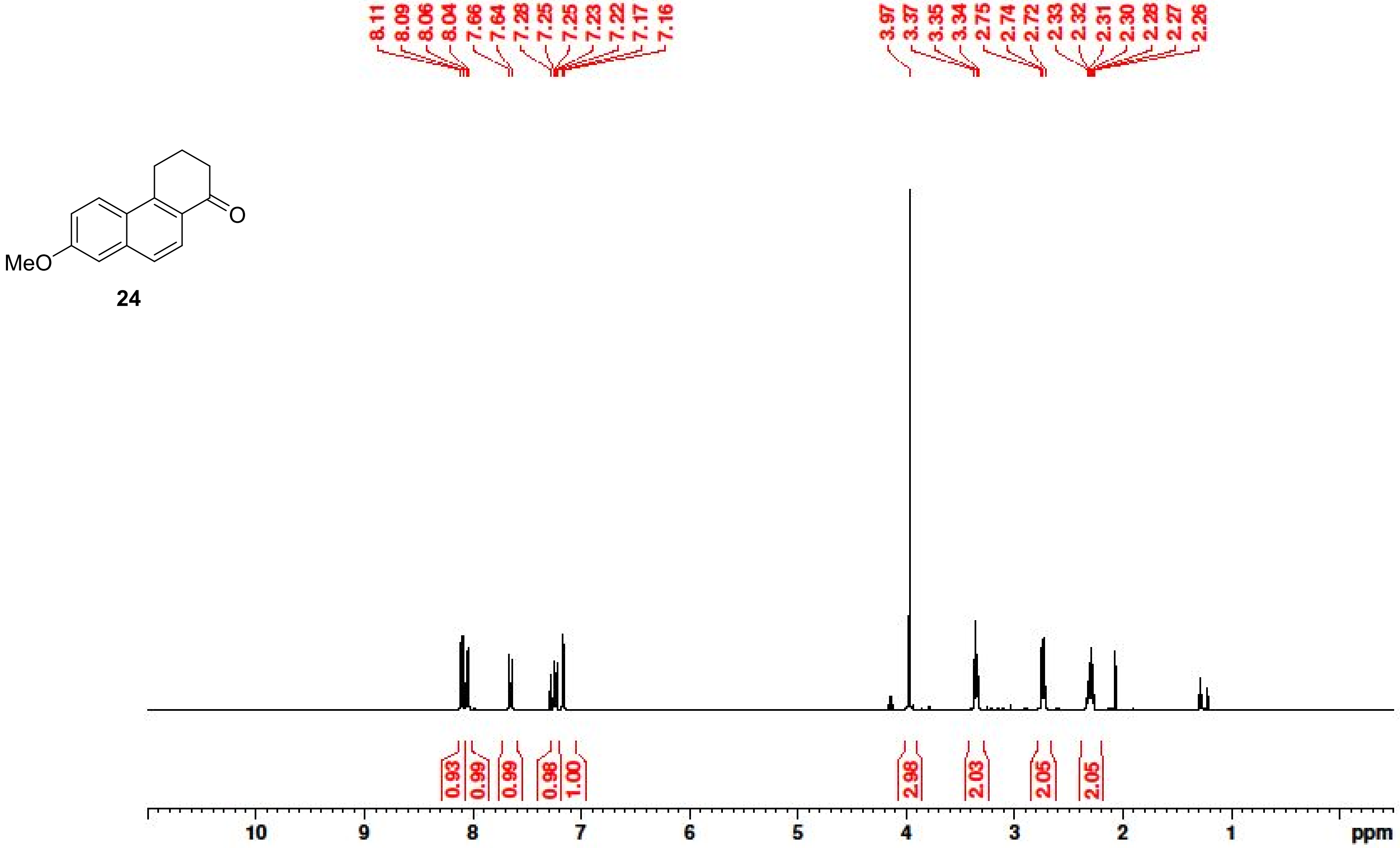

Figure S17. ^{1}H NMR spectrum of **24**

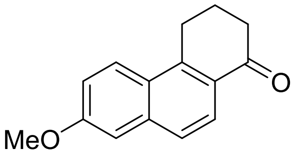

24

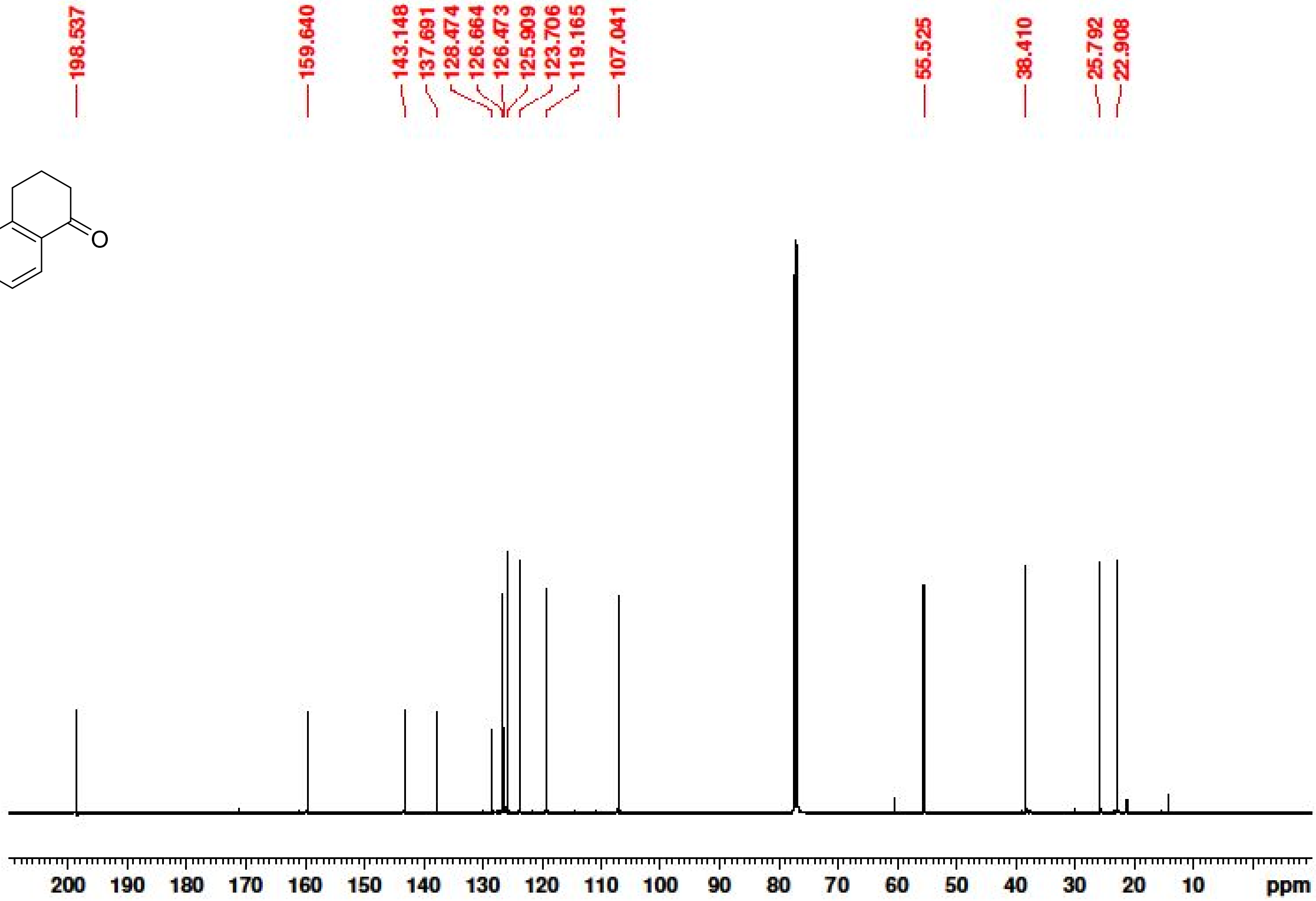

Figure S18. $^{13}C\{^{1}H\}$ NMR spectrum of **24**

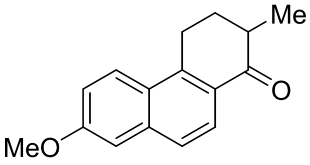

9

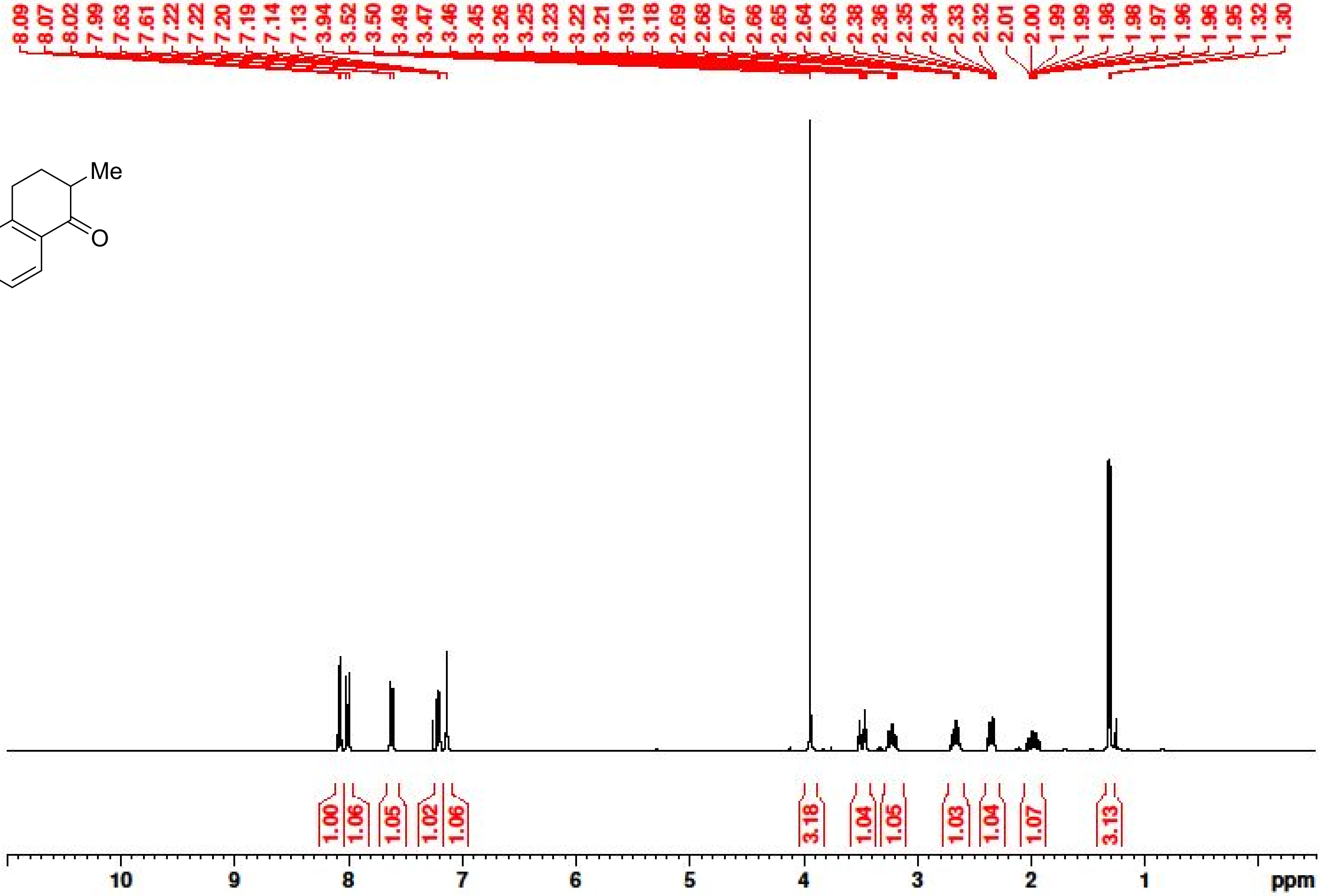

Figure S19. ^{1}H NMR spectrum of **9**

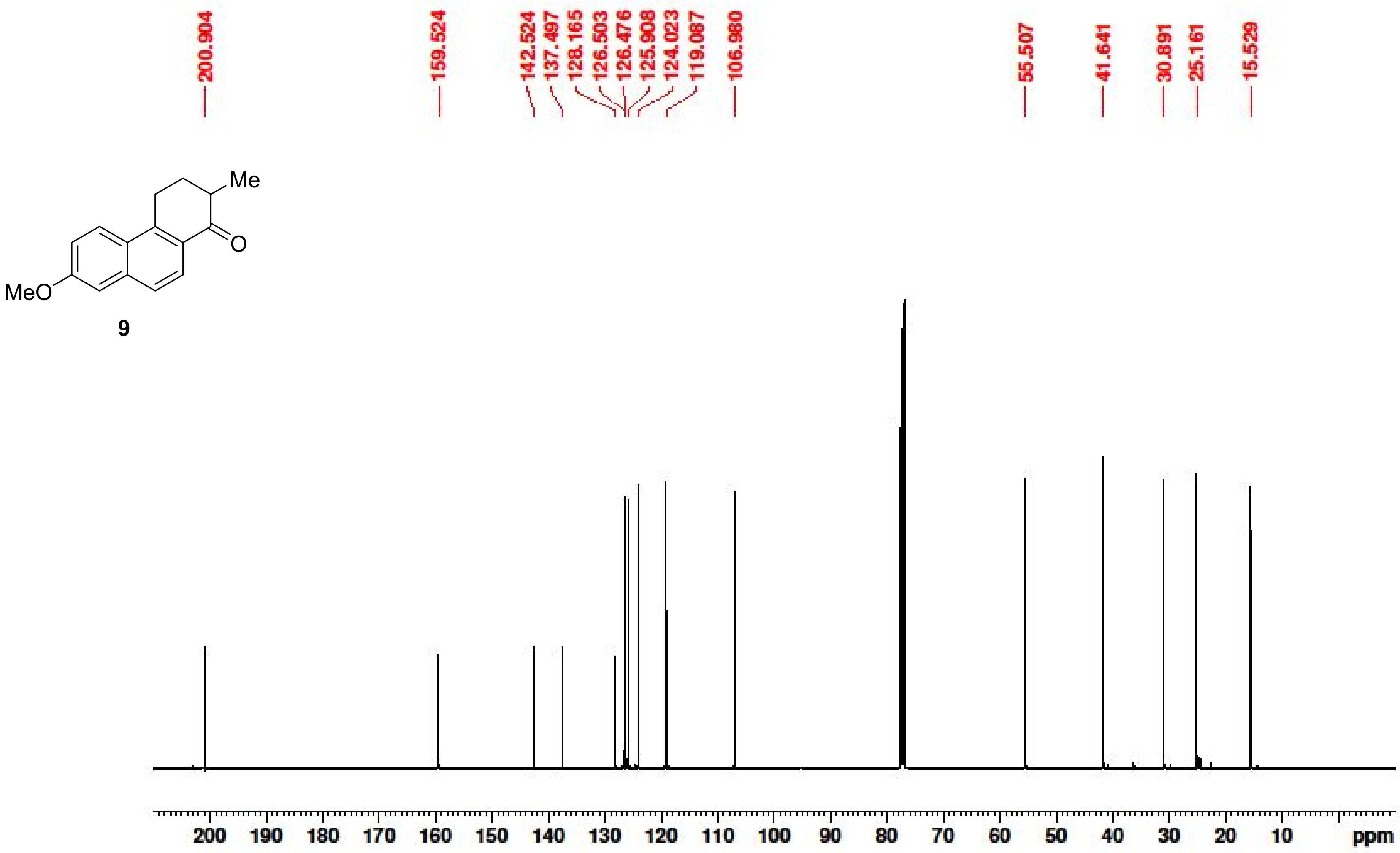

Figure S20. $^{13}C\{^{1}H\}$ NMR spectrum of **9**

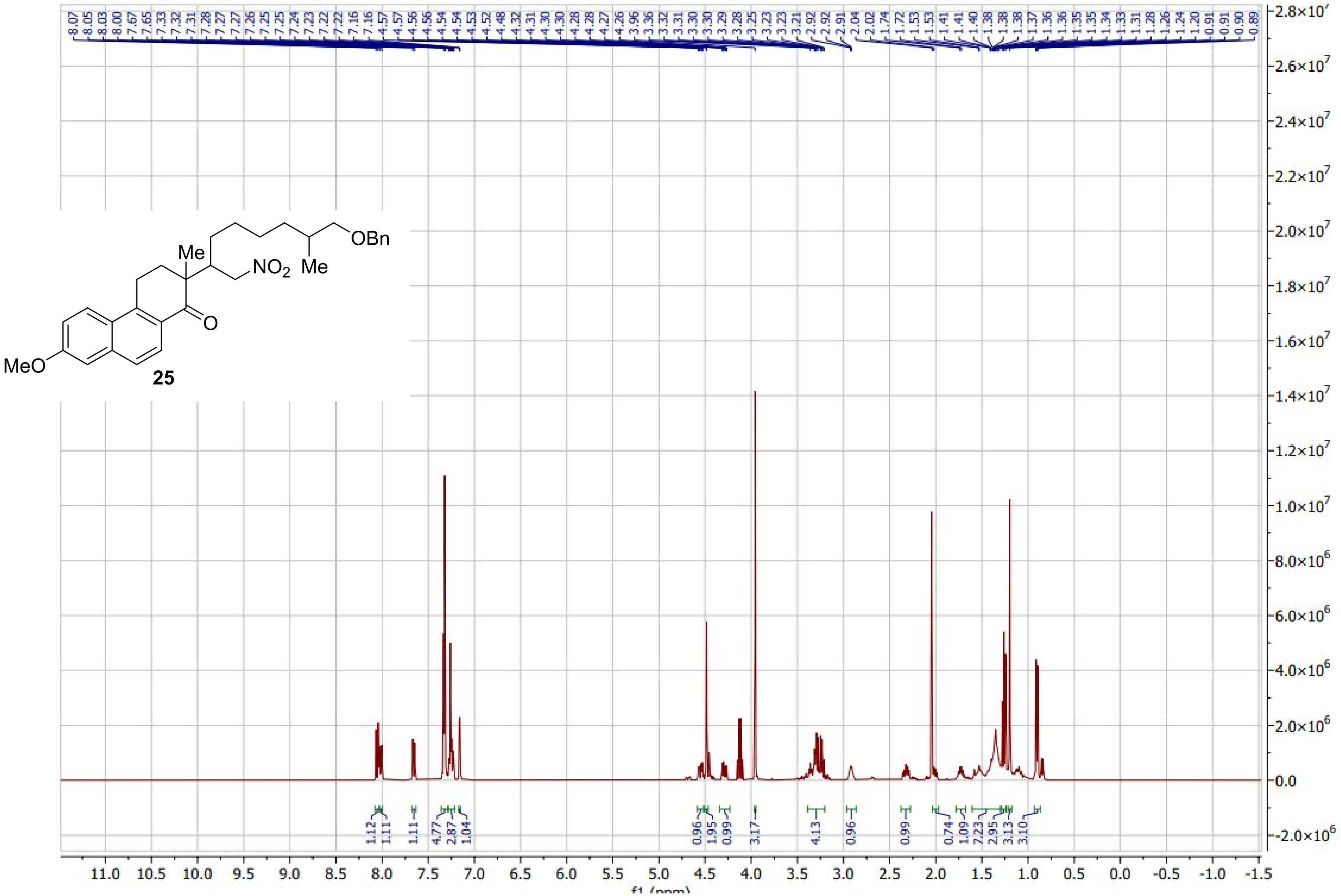

Figure S21. ^{1}H NMR spectrum of **25**

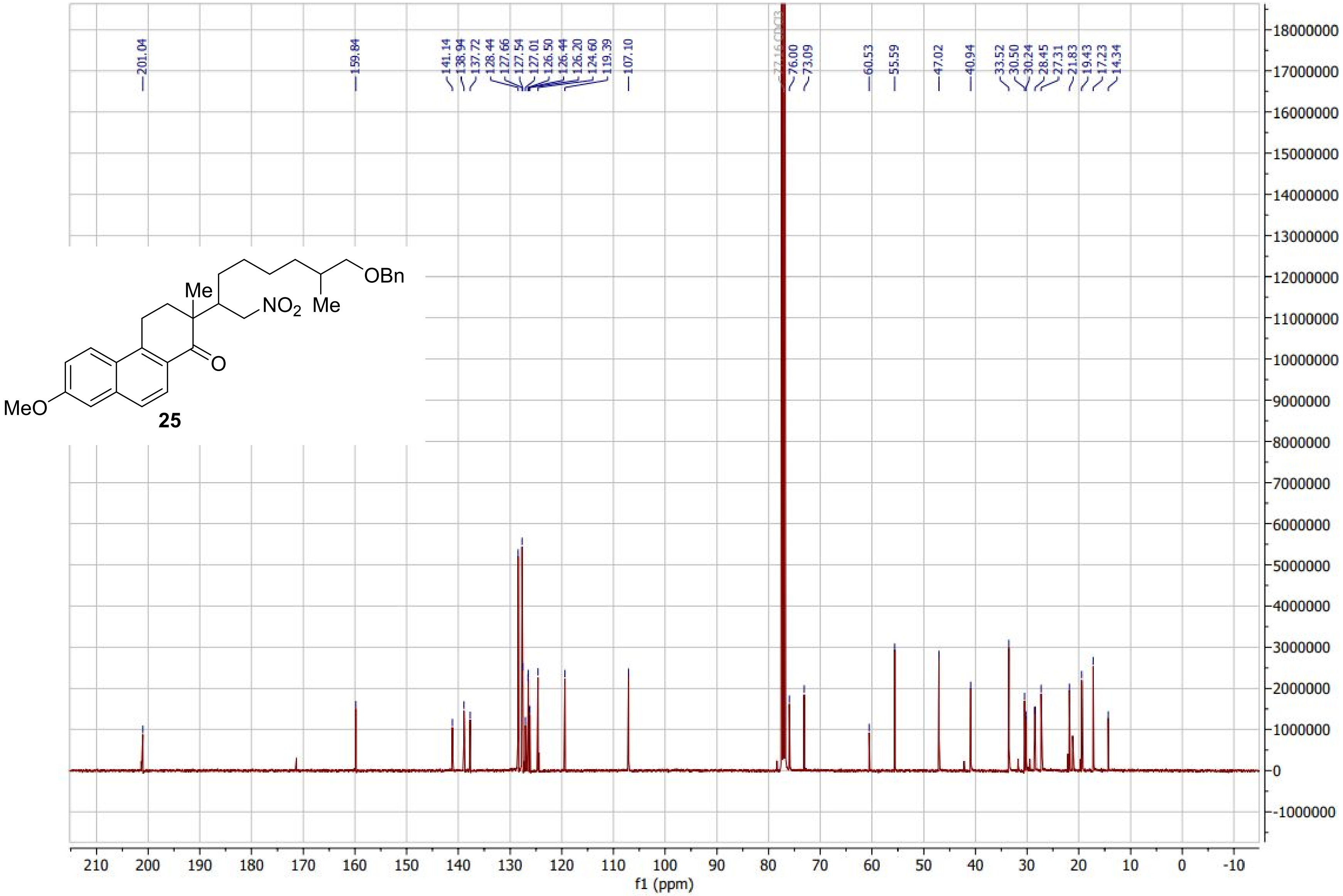

Figure S22. $^{13}C\{^{1}H\}$ NMR spectrum of **25**

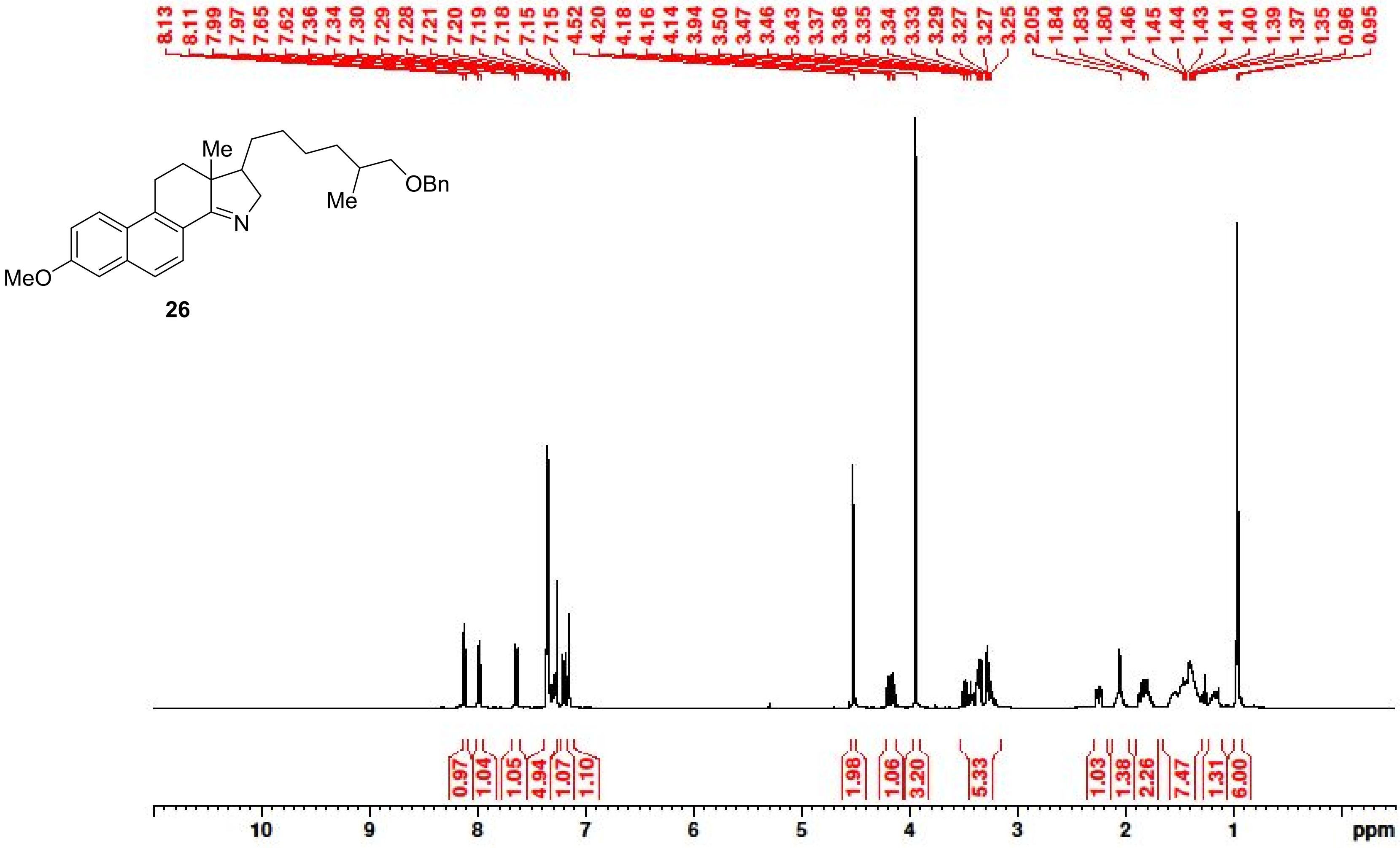

Figure S23. ^{1}H NMR spectrum of **26**

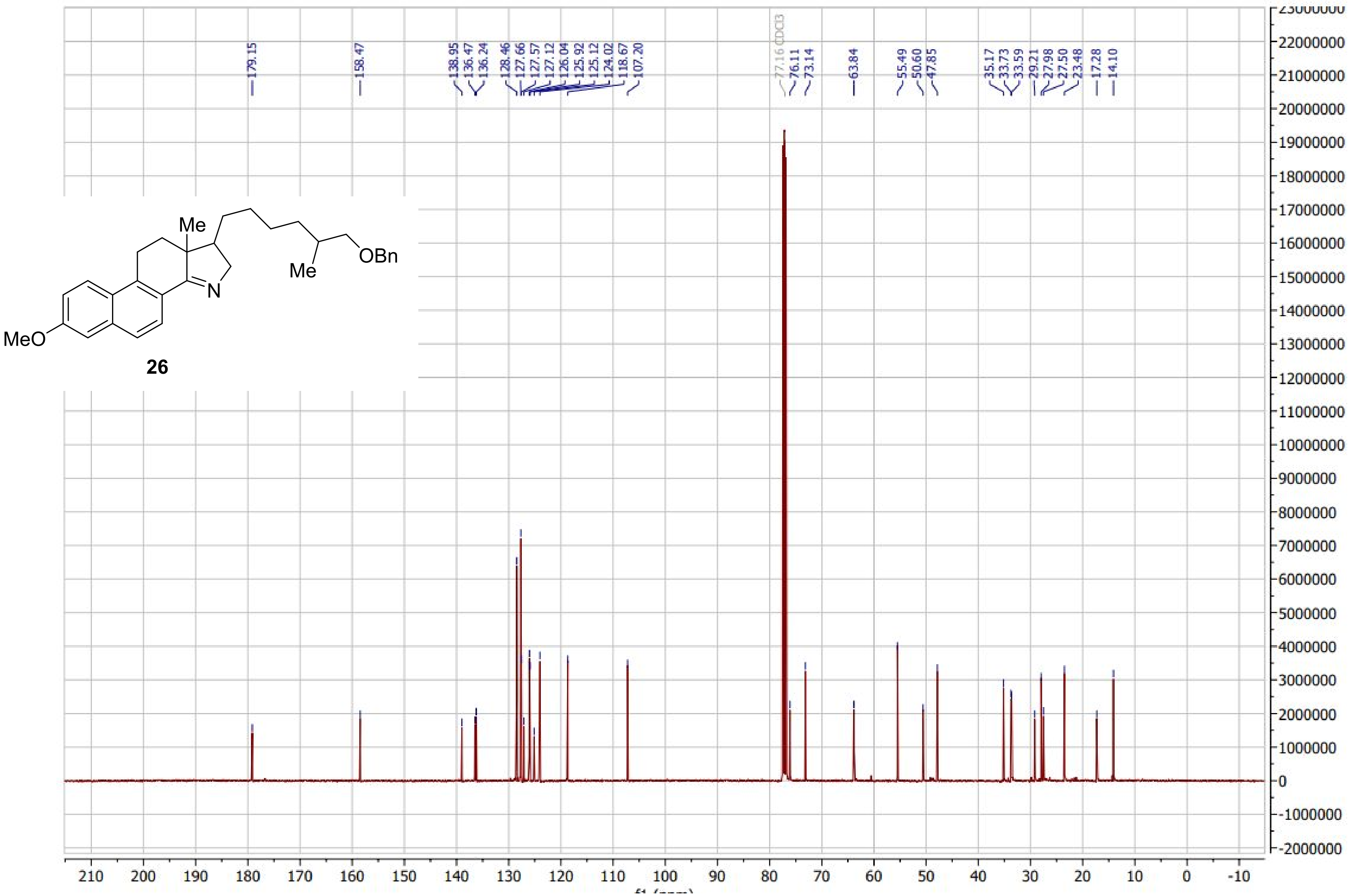

Figure S24. $^{13}C\{^{1}H\}$ NMR spectrum of **26**

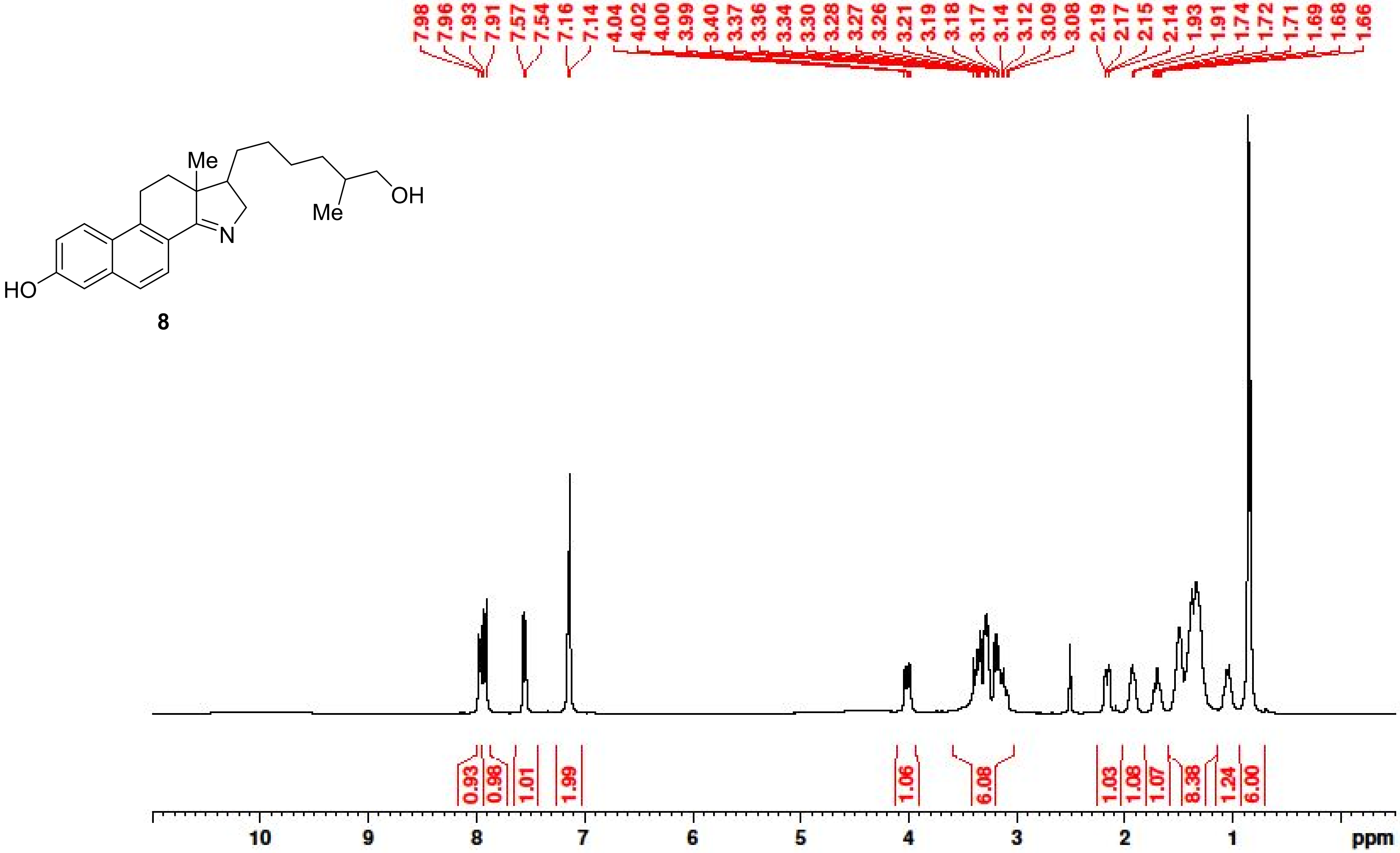

8

Figure S25. ^{1}H NMR spectrum of **8**

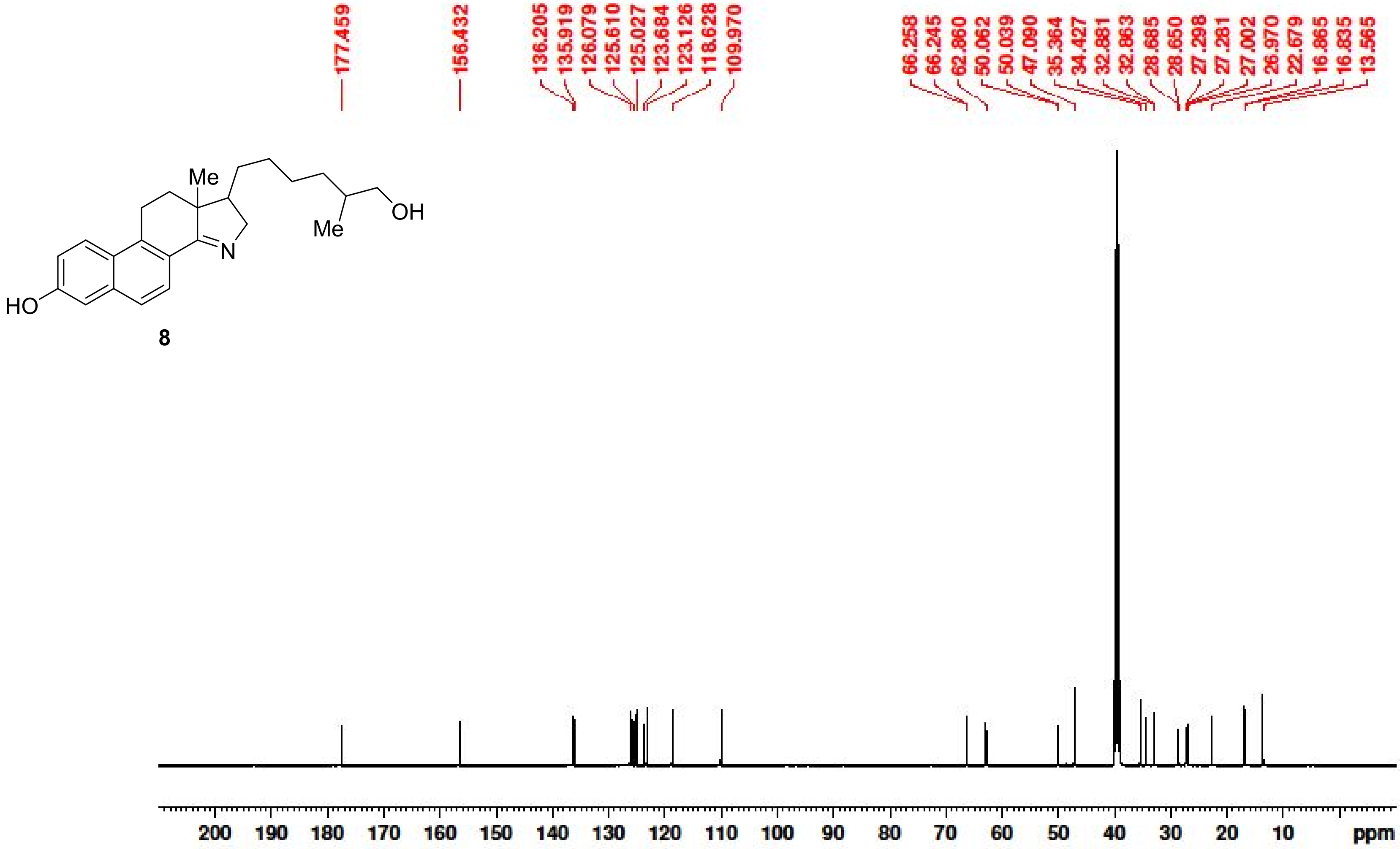

Figure S26. $^{13}C\{^1H\}$ NMR spectrum of **8**

Analytical Chiral SFC Chromatogram of 8

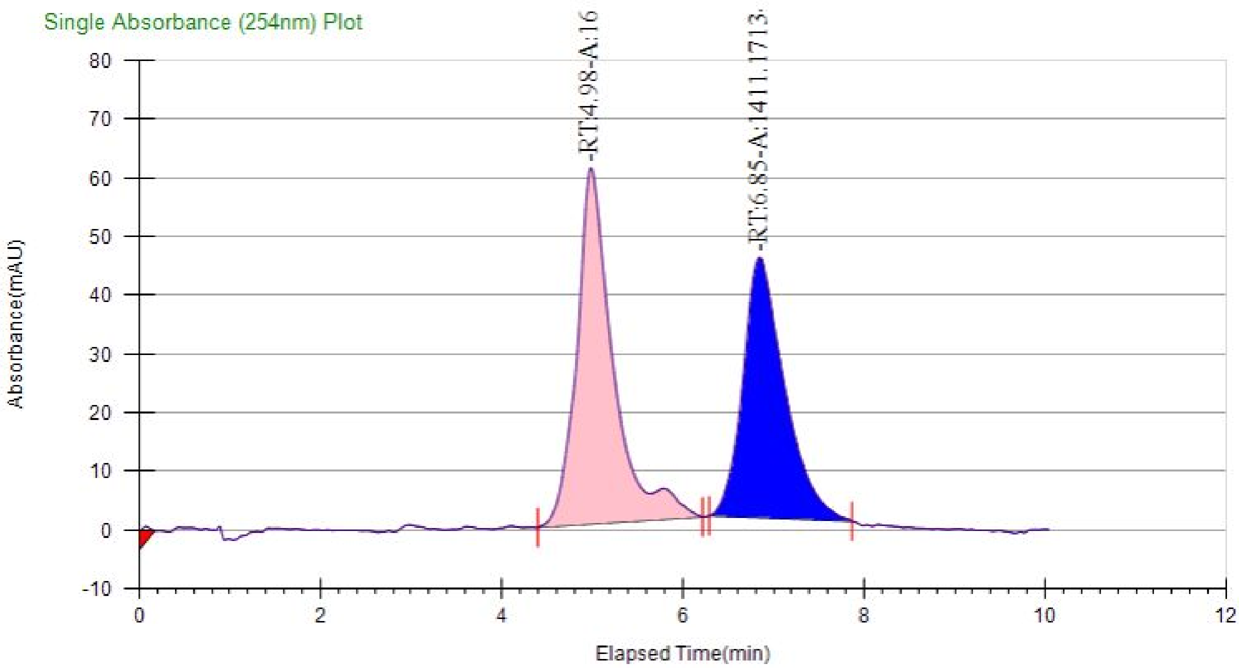

General Information

Log Author	Log Date	Report By	Report Date	Notes
Administrator	12/21/2021 9:06:51 AM	Administrator	12/22/2021	

Run Information

Instrument Method	Inj. Vol.	Solvent	Column	Sample	Well Location	Temp.	Flow	% Modifier	Pressure
15 Isocratic	25	1: MeOH (0.1%DEA)	Lux Cellulose-3 (4.6 x 250mm)	VU6054831 test	15C	40	3.5	15	100

Peak Information

Peak No	% Area	Area	Ret. Time	Height	Cap. Factor
1	54.522	1691.8043	4.98 min	60.7085	0
2	45.478	1411.1713	6.85 min	44.3695	0

Figure S27: Analytical Chiral SFC of 8
